# Epigenetic dynamics shaping melanophore and iridophore cell fate in zebrafish

**DOI:** 10.1186/s13059-021-02493-x

**Published:** 2021-10-04

**Authors:** Hyo Sik Jang, Yujie Chen, Jiaxin Ge, Alicia N. Wilkening, Yiran Hou, Hyung Joo Lee, You Rim Choi, Rebecca F. Lowdon, Xiaoyun Xing, Daofeng Li, Charles K. Kaufman, Stephen L. Johnson, Ting Wang

**Affiliations:** 1 Department of Genetics, Washington University School of Medicine, St Louis, MO USA; 2 The Edison Family Center for Genome Sciences and Systems Biology, Washington University School of Medicine, St. Louis, MO USA; 3 Present address: Department of Epigenetics, Van Andel Institute, Grand Rapids, MI USA; 4 Department of Medicine, Division of Medical Oncology, and Department of Developmental Biology, Washington University in Saint Louis, St. Louis, MO USA; 5 McDonnell Genome Institute, Washington University School of Medicine, St. Louis, MO USA

## Abstract

**Background:**

Zebrafish pigment cell differentiation provides an attractive model for studying cell fate progression as a neural crest progenitor engenders diverse cell types, including two morphologically distinct pigment cells: black melanophores and reflective iridophores. Nontrivial classical genetic and transcriptomic approaches have revealed essential molecular mechanisms and gene regulatory circuits that drive neural crest-derived cell fate decisions. However, how the epigenetic landscape contributes to pigment cell differentiation, especially in the context of iridophore cell fate, is poorly understood.

**Results:**

We chart the global changes in the epigenetic landscape, including DNA methylation and chromatin accessibility, during neural crest differentiation into melanophores and iridophores to identify epigenetic determinants shaping cell type-specific gene expression. Motif enrichment in the epigenetically dynamic regions reveals putative transcription factors that might be responsible for driving pigment cell identity. Through this effort, in the relatively uncharacterized iridophores, we validate *alx4a* as a necessary and sufficient transcription factor for iridophore differentiation and present evidence on *alx4a*’s potential regulatory role in guanine synthesis pathway.

**Conclusions:**

Pigment cell fate is marked by substantial DNA demethylation events coupled with dynamic chromatin accessibility to potentiate gene regulation through cis-regulatory control. Here, we provide a multi-omic resource for neural crest differentiation into melanophores and iridophores. This work led to the discovery and validation of iridophore-specific *alx4a* transcription factor.

**Supplementary Information:**

The online version contains supplementary material available at 10.1186/s13059-021-02493-x.

## Background

The development of a multicellular organism is an intricate process of expansion and diversification of a pluripotent cell population. Rapidly following embryogenesis, the genome of stem cells experiences extensive biochemical and structural changes that allow these multipotent progenitor cells to faithfully commit and differentiate into various tissue and cell types. These decisions are often reflected by unique gene expression profiles and are shaped by epigenetic programs [1, 2]. Although monumental consortium level efforts, such as ENCODE [3] and Roadmap Epigenomics [4], have significantly advanced the field of developmental epigenetics, these studies have mostly focused on profiling human and mouse model systems.

Zebrafish neural crest cells (NCCs) differentiate into various morphologically and functionally distinct cell types, such as glia, neurons, cartilage, connective tissue and pigment cells [5]. How a single-cell population with the same genetic content could generate such diverse cell types is an active field of research in developmental biology. Zebrafish have three main pigment cell types, black melanophore, reflective iridophore, and yellow xanthophore, which are all derived from a multipotent neural crest cell population [5–10]. Various mutagenesis experiments in zebrafish provided insights into the genetic regulation and gene regulatory networks responsible for pigment cell differentiation [11–15]. Melanophore development has been extensively studied for its translational potential in tackling melanoma. In melanophores, *sox10* [16] and Wnt signaling [17] are required to activate and stabilize expression of *mitfa*, which is an essential transcription factor regulating numerous melanophore differentiation genes, including those controlling melanin synthesis [18]. Although relatively understudied, a few molecular mechanisms governing iridophore cell fate have been discovered in forward genetic screens. In iridophore development, *pnp4a* [19] was shown to encode an enzyme important in the biosynthesis of guanine, an important molecule responsible for the reflective characteristic in iridophores. Furthermore, PKA (protein kinase A) signaling [20], Alk (Anaplastic lymphoma kinase), and Ltk (leucocyte tyrosine kinase) ligands [21] are essential for iridophore development. The gene regulatory network for iridophore differentiation is underexplored. However, *sox10* [10], *foxd3* [9, 22], *tfec* [23], and *gbx2* [24] transcription factors have been implicated in iridophore cell fate.

Although forward genetic experiments offered valuable mechanistic insights [10–14], a systematic description of the underlying gene regulatory network for pigment cell differentiation is still lacking. In this study, we highlight how comparative epigenetics can be a powerful tool in deciphering both the genetic and epigenetic mechanisms that govern cell fate. Here, we provide some of the first insights into the epigenetic dynamics that shape neural crest differentiation into pigment cells in zebrafish by providing high-quality epigenetic landscape profiles of various stages of NCC differentiation into melanophores and iridophores. In conclusion, we leverage DNA methylation and chromatin accessibility dynamics to chart putative gene regulatory networks that govern pigment cell fate to discover that *alx4a* is necessary and sufficient for iridophore development on the zebrafish body.

## Results

### Neural crest and pigment data collection and generation

**Fig. 1:**
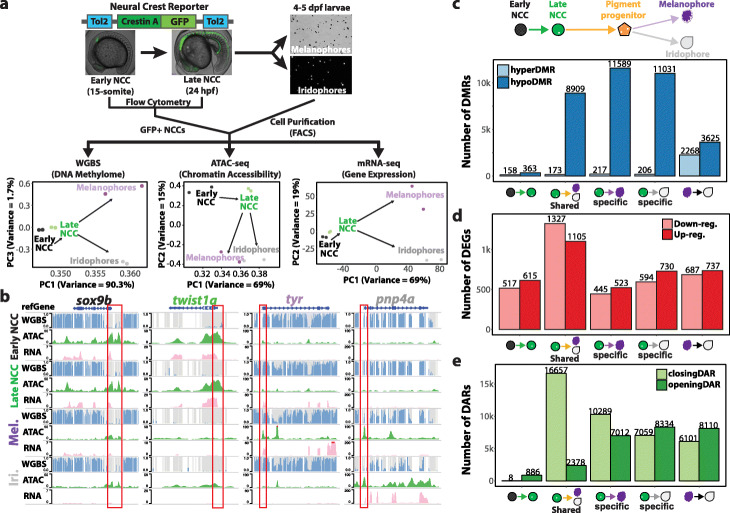
Epigenetic and transcriptomic dynamics of neural crest cell differentiation into pigment cells. **a** Schematic of sample collection method and principle component analysis on the DNA methylome, chromatin accessibility, and transcriptome of each cell type. **b** WashU Epigenome browser views of zebrafish early neural crest marker gene (*sox9b*), neural crest marker gene (*twist1a*), melanophore marker gene (*tyr*), and iridophore marker gene (*pnp4a*). Grey bars represent CpG sites while height of the blue bars indicates methylation levels. The red box demarcates promoter regions of marker genes. **c–e** Bar charts illustrating the number of DMRs (**c**), DEGs (**d**), and DARs (**e**) when comparing early NCCs, late NCCs, melanophores, and iridophores. DMRs were identified using DSS (*p* value < 0.001)

To capture the DNA methylation, chromatin accessibility, and gene expression landscapes during pigment cell differentiation, we generated two biological replicates of whole-genome bisulfite sequencing (WGBS), assay for transposase-accessible chromatin using sequencing (ATAC-seq), and mRNA-seq libraries respectively. The zebrafish *crestin* gene can serve as a marker for neural crest specification and migration during zebrafish embryogenesis [25]. Therefore, we created a transgenic fish with a *crestin* promoter driving GFP expression and isolated GFP-positive NCCs from 15-somite and 24 h post-fertilization (hpf) embryos (Fig. 1a). These two time points reflect the onset [25] and partially committed states [26] of neural crest cells respectively. We also included the fully committed states by isolating melanophores and iridophores from 4 to 5 days post-fertilization (dpf) larvae [27].

These isolation techniques generated reproducible and biologically diverse samples as reflected by the principal component analysis (PCA) on each genomic assay (Fig. 1a). The biological replicates clustered closely together, while cells from different stages along the NCC development trajectory were separated from one another. To further verify that we collected and profiled the correct cell types, we examined well-established marker genes *sox9b* [28], *twist1a* [29], *tyr* [30], and *pnp4a* [9], which represent early NCCs, late NCCs, melanophores, and iridophores respectively. Indeed, these marker genes are highly expressed and the promoters of these marker genes (red box, Fig. 1b) show low methylation and high chromatin accessibility in specific cell types.

### Focal mCpG loss demarcates cell identity

We first focused on DNA methylation changes considering its association with establishing cell identities (Additional file 1: Fig. S1a-f, Additional file 2: Table S1). Following the NCC to pigment cell differentiation path, we found a slight decrease in the global DNA methylation levels (~ 85 to ~ 81%, Additional file 1: Fig. S1g). We utilized DSS tool (Methods, *p* value < 0.01) to identify thousands of differentially methylated regions (DMRs) (Fig. 1c, Additional file 1: Fig. S1h) and discovered that pigment cell differentiation is accompanied by a largely focal loss of methylation and very minimal gain of methylation (Fig. 1c). We also note that melanophores and iridophores share regions that undergo similar magnitude of methylation change (Additional file 1: Fig. S1i) from 24 hpf NCC. Differentially expressed genes near these shared hypoDMRs enrich for GO terms related to neural crest migration (“ameboidal-type cell migration”) [31] and pigmentation (Additional file 1: Fig. S1j), suggesting that DNA methylation could play a role in early phases of neural crest differentiation into pigment cells.

### Chromatin accessibility tunes transcription

Although the loss of DNA methylation at promoters or enhancers are often associated with gene activation [32], we report relatively balanced gene expression dynamics during pigment cell differentiation where hundreds of genes are up- or downregulated (Fig. 1d) as identified by DESeq2 (Methods, adj. *p* value < 0.01). Therefore, we hypothesized that chromatin accessibility might be playing a potential role in epigenetic suppression of gene activity. Using DiffBind (Methods, FDR < 0.001), we identified distinct and shared chromatin accessible regions across the samples (Additional file 1: Fig. S2a-d). We report more than twice as many closing differentially accessible regions (DARs) than opening DARs in both melanophores and iridophores during pigment differentiation (Fig. 1e). These data suggest that although the majority of the DNA methylation dynamics favor epigenetic activation, the chromatin accessibility could be important for fine-tuning the cell-type-specific epigenetic suppression. Furthermore, when we focused on iridophore-specific chromatin accessibility dynamics, we discovered that super-accessible regions (Methods) often demarcated genes that were highly expressed in iridophores, such as *pnp4a* (Additional file 1: Fig. S2e,f). These regions could provide a superenhancer-like mechanism to drive cell fate decisions [33], but warrant further investigation.

### DMARs as putative cis-regulatory elements

**Fig. 2:**
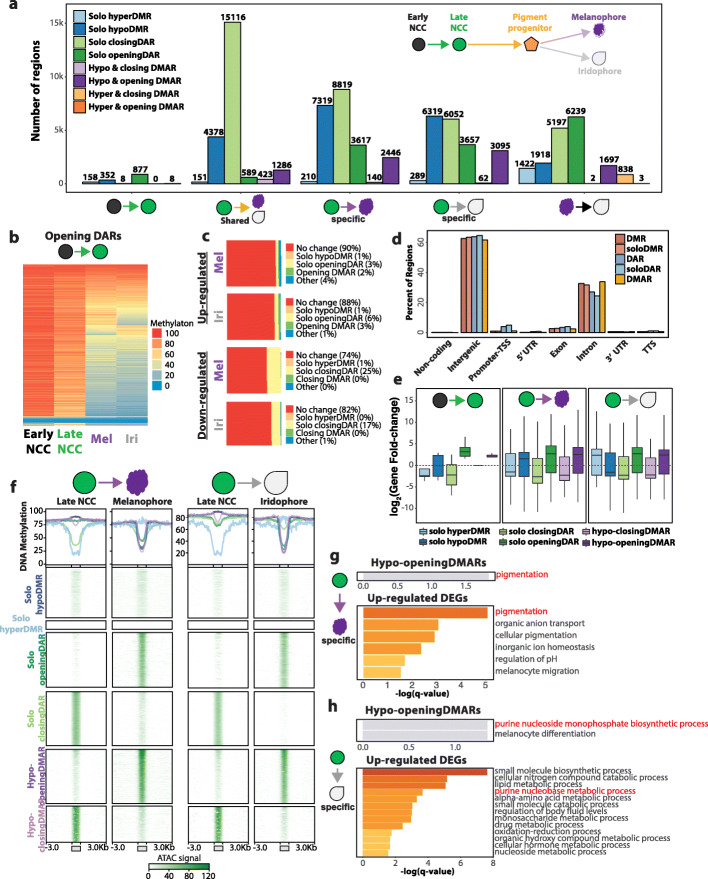
Characterization and annotation of DMARs. **a** Bar plot illustrating the number of DMARs identified across pigment cell differentiation. No HyperDMARs were detected, except in melanophore vs. iridophore comparison. **b** Heatmap illustrating the DNA methylation levels of opening DARs identified in early NCC to late NCC transition. **c** Epigenetic dynamics of DEG promoters in melanophores and iridophores. **d** Genomic feature distribution of DMRs, DARs, and DMARs. **e** Expression fold-change of closest DEGs within 50 kb of epigenetically dynamic regions. **f** Line graphs and heatmaps representing average DNA methylation levels and ATAC peak signals respectively of epigenetically dynamic regions from late NCC to pigment cell-type comparison. **g, h** Gene ontology enrichment of DEGs within 50 kb of hypo-opening DMARs and upregulated DEGs in melanophore-specific (**g**) and iridophore-specific (**h**) comparison

DNA methylation and chromatin accessibility dynamics can collaboratively influence epigenetic control. Therefore, we characterized various combinations of dynamics of differential methylations and accessibilities (Additional file 1: Fig. S3a, Additional file 2: Table S2). We report thousands of shared and cell-type-specific differentially methylated and accessible regions (DMARs) that define pigment cell differentiation (Fig. 2a). The majority of DMARs have their size and CpG density fall in similar distribution as DMRs and DARs alone (Additional file 1: Fig. S3b,c). DMRs, DARs, and DMARs associate with higher phastCons [34] and phyloP [35] conservation scores, suggesting that these regions might be evolutionarily preserved with potential functional consequences (Additional file 1: Fig. S3d). We report that majority of the epigenetic dynamics that define the transition from early NCC to late NCC is increased chromatin accessibility. Interestingly, these opening DARs were highly methylated in late NCC but subsequently lose methylation in melanophores and iridophores (Fig. 2b). This could suggest that certain subpopulations of neural crest cells have pigment enhancers already primed or marked by chromatin accessibility that only become functional after subsequent DNA demethylation later during differentiation.

Next, we explored promoter epigenetic status of differentially expressed genes (DEGs). Roughly 88–90% of promoters of upregulated genes are epigenetically static from 24 hpf NCC to pigment cell differentiation (Fig. 2c). Although a small fraction of promoters of downregulated genes might be repressed by loss of accessibility, the majority does not experience any epigenetic change. This result suggests that gene expression is more likely to be controlled by DMRs, DARs, or DMARs in enhancer context. Indeed, majority of these regions are in intergenic or intronic regions (Fig. 2d), which if epigenetically active, can play a cis-regulatory role. We also report that DEGs close to openingDARs and hypo-openingDMARs show increase in expression while DEGs near closingDARs and hypo-closingDMARs trended to lose expression further supporting the cis-regulatory potential of these epigenetically dynamic regions (Fig. 2e). Interestingly, solo DMRs near DEGs were not as predictive of the gene expression change as DARs or DMARs. Further look into these solo DMRs revealed that 65% and 71% of solo hypoDMRs in melanophores and iridophores did not contain ATAC-seq peaks (Additional file 1: Fig. S4a). The solo hypoDMRs show relatively low ATAC signal, have lower CpG count, and are smaller in size compared to DMARs (Fig. 2f, Additional file 1: Fig. S4b). These insights imply that these solo hypoDMRs are no longer accessible to provide cis-regulatory function in differentiated pigment cells. In context of solo DARs, 43% and 15% of solo closingDARs and 22% and 40% of solo openingDARs occur in regions with < 30% DNA methylation in melanophores and iridophores respectively (Additional file 1: Fig. S4a,c,d), highlighting the fine-tuning that chromatin accessibility provides for gene regulation at later stages of pigment cell differentiation.

To explore the potential biological cis-regulatory role of the DMRs, DARs, and DMARs, we performed gene ontology (GO) enrichment of DEGs nearby these epigenetically dynamic regions (Fig. 2g–h, Additional file 1: Fig. S5a-d). GO enrichment of DEGs near hypoDMRs and solo closingDARs converge on terms related to central nervous system and embryonic development. These terms are also present in the GO enrichment of downregulated gene from early NCC to late NCC to pigment cells (Additional file 1: Fig. S6a,c-e), which suggests that DNA methylation plays a role in early neural crest development that becomes silenced through closing of chromatin. Similar enrichment analysis of upregulated DEGs presented analogous annotation enrichments of that for shared openingDARs and hypo-opening DMARs (Fig. 2g,h, Additional file 1: Fig. S5d). For example, melanophore-specific genes reflect pigmentation while iridophore-specific genes enrich for purine synthesis that is responsible for guanine crystal stacks that give iridophore its reflective properties.

### Defining the transcription factor network of pigment cell differentiation

**Fig. 3:**
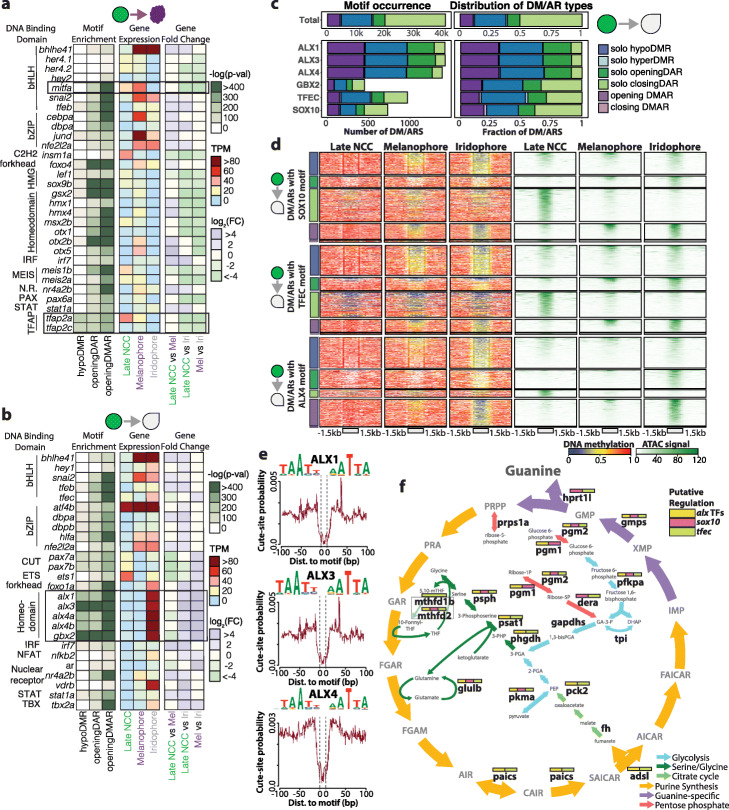
Motif enrichment analysis reveals alx transcription factor family as putative regulator of iridophore development. **a**, **b** Heatmaps representing motif enrichment, gene expression, and gene fold change of transcription factors when comparing late NCC differentiation into melanophores (**a**) and iridophores (**b**). **c** Bar plots representing frequency and distribution of iridophore-associated DM/ARs with a particular TF motif. **d** Heatmaps representing DNA methylation and ATAC signal across iridophore-associated DM/ARs with specific TF motifs. **e** ATAC-seq footprint signatures of alx transcription factor candidates. **f** Model of guanine synthesis cycle. Iridophore-specific DEGs are shown in bold. Boxes above DEGs are color-coded based on detection of CREs containing TF motifs within 50 kb of DEG promoters

The epigenetic landscape is often intricately tied with transcription factor (TF) presence and binding events [36, 37]. We performed motif enrichment analysis in melanophore-specific or iridophore-specific DMRs, DARs, and DMARs (Additional file 2: Table S3-6) to identify the potential roles of pigment cell-specific TFs. We identified motif enrichment of known regulators for the differentiation of melanophore (TFAP-related motifs and MiT/TFE motifs) and iridophore (*tfec*) [38] (Fig. 3a), as well as that of novel TF candidates: *hey1*, *ets1*, *foxo1a*, *nfkb2*, *ar*, *vdrb*, *tbx2a*, *gbx2*, and aristaless homeobox TFs (*alx1*, *alx3*, *alx4a*, and *alx4b)* (Fig. 3b)*.* Among these newly discovered candidates, aristaless homeobox TFs showed most significant enrichment, suggesting their importance in iridophore differentiation. Indeed, when we quantified the number of DM/ARs with ALX, GBX2, TFEC, and SOX10 motifs, transcription factors deemed to be important for iridophore differentiation, we saw that the ALX-containing DM/ARs were most abundant (Fig. 3c). In addition, ALX-containing DM/ARs were over-represented by hypo-openingDMARs (~ 3 folds greater than expected) and solo openingDARs (~ 2.2 folds greater than expected) while under-represented by solo closingDARs (~ 5.7 folds less than expected). In comparison, GBX2-containing, TFEC-containing, and SOX10-containing hypo-opening DMARS were only ~ 1.7, ~ 1.5, and ~ 1.9 folds greater than expected respectively. Next, we explored the DNA methylation and chromatin accessibility dynamics associated with the iridophore-associated epigenetically dynamic regions with ALX, SOX10, GBX2, and TFEC motifs. This analysis led to the discovery that a fraction of solo openingDARs and hypo-openingDMARs that contain SOX10 (17.2 and 29.5%), TFEC (19.6 and 41.5%), and GBX2 (17.6 and 17.9%) motifs were shared in the melanophore (Fig. 3d, Additional file 1: Fig. S7a). In stark contrast, less than 6% and 14% of the solo openingDARs and hypo-openingDMARs containing the ALX4 motif respectively were present in melanophores. Considering that ALX-containing DM/ARs are most frequent and enrich for activating epigenetic dynamics (openingDAR or openingDMAR) along with the discovery that majority of the ALX-containing DMARs are specific to iridophores suggests the potential importance that the *alx* transcription factors might have to iridophore cell fate. Furthermore, analysis of iridophore ATAC peaks revealed strong footprinting signatures for these aristaless homeobox and other TF candidates, suggesting their binding activity (Fig. 3e, Additional file 1: Fig. S7b).

To understand how TFs control gene regulatory networks crucial for iridophore biology and identity, we first examined whether iridophore TFs are turned on and regulated during iridophore differentiation from NCC. When we scanned for epigenetically activating DMRs and/or DARs (DM/ARs) within 50 kb of iridophore-specific TF promoters, we discovered that the *alx4a* promoter had one of the highest DM/AR occurrences (17 DM/ARs, 14 with iridophore-related TF motifs) (Additional file 1: Fig. S7c,d). We were encouraged by this discovery as *alx4a* was recently discovered to be a novel iridophore marker through single-cell expression analysis [39]. This result suggests the robust epigenetic activation of *alx4a* in iridophores, but not melanophores. By leveraging DM/ARs and motif presence near important iridophore TFs, we constructed a putative transcription factor network that drives iridophore cell fate (Additional file 1: Fig. S7d). Next, we focused on genes in the guanine synthesis pathway, which is crucial for iridophore physiology [27]. Numerous iridophore-specific DEGs (70.6%) responsible for guanine generation and transport have at least one DMAR with *alx* motif (Fig. 3f, Additional file 1: Fig. S8). Furthermore, we looked at DEGs in the top four GO enrichment categories that had DM/ARs within 50 kb of their promoters. In total, 76% of these DEGs have at least one DM/AR with an *alx* motif (Additional file 1: Fig. S8), suggesting the putative regulatory potential of *alx* TFs for iridophore’s reflective characteristic.

### alx4a TF is essential for iridophores

**Fig. 4:**
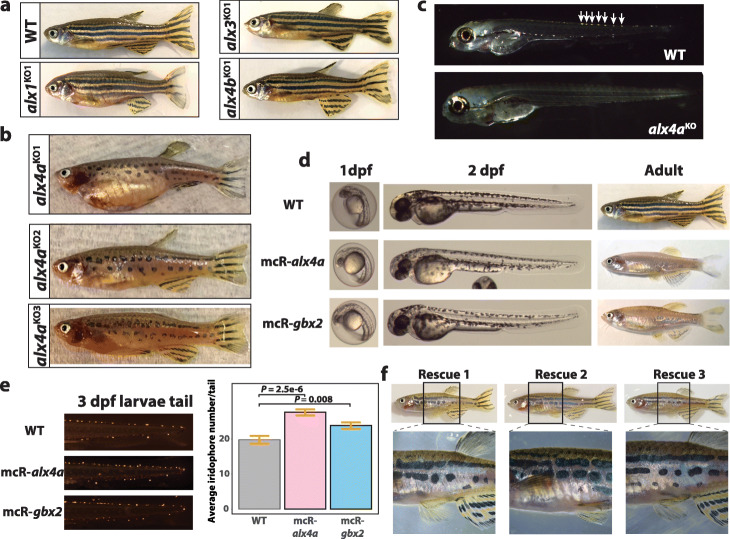
Functional validation of alx4a and gbx2 in iridophore development. **a** Lateral view of WT and alx1KO, alx3 KO, alx4b KO fish. **b** Lateral view of alx4a CRISPR-mediated knockout fish. **c** Iridophore detection in 4 dpf larvae of WT and alx4a knockout larvae. White arrows mark iridophores in WT larvae. **d** Lateral views of 1 dpf larvae, 2 dpf larvae, and adult fish comparing WT to Tg(miniCoopR-alx4a) and Tg(miniCoopR-gbx2) fish. **e** Representative pictures and quantification of iridophores from 3 dpf larvae tail trunks of WT (*n* = 21), Tg(miniCoopR-alx4a) (*n* = 20), and Tg(miniCoopR-gbx2) (*n* = 20). *P* values were calculated with two-tailed Welch’s *t*-test. Error bars represent ± SE. **f** Lateral whole-body view of iridophore rescue in three mosaic tg(miniCoopR-alx4a;alx4aKO) fish. Black box denotes the zoomed region in picture below

Recently, the knockdown of *gbx2* through morpholino experiments has been shown to diminish iridophore count in zebrafish larvae [24]. However, whether the *alx* TFs are necessary for iridophore cell fate is currently unknown. Therefore, we utilized CRISPR-Cas9 technology to generate stable *alx* knockout fish. In *alx1*^KO^, *alx3* ^KO^, and *alx4b* ^KO^ fish, however, iridophores develop normally with only some instances of pigment pattern defect in the caudal fin, suggesting that TFs from the same family might not be necessary for iridophore differentiation (Fig. 4a, Additional file 1: Fig. S9). We report that with the exception of eyes, *alx4a*^KO^ fish revealed complete ablation of iridophores, similar to *shady*, *rse*, and *tra* mutant fish [40, 41]. In 4 dpf *alx4a*^KO^ larvae, no iridophores are present suggesting that iridophores fail to differentiate when *alx4a* is absent (Fig. 4c). The presence of iridophores in the eye suggests that an alternative gene regulatory network is responsible for eye iridophore differentiation, analogous to the complimentary roles of *otx* and *mitfa* in melanophore differentiation in the eye and body, respectively [42].

### alx4a biases towards iridophore fate

Since *alx4a* and *gbx2* are essential for proper iridophore development, we asked whether either TF was sufficient to push pigment cell fate towards iridophores. To ectopically express *alx4a* and *gbx2* in early pigment progenitor cells, we utilized the *mitfa* promoter in the miniCoopR transgenesis system [43, 44] (Additional file 1: Fig. S10a,b). We note that 2 dpf transgenic larvae show embryonic melanophores, but almost no melanophores at adult stage, reminiscent of *nacre* (*mitfa* mutant) fish [15] (Fig. 4d, Additional file 1: Fig. S10c). Furthermore, 3 dpf larvae of *Tg(miniCoopR-alx4a)* and *Tg(miniCoopR-gbx2)* fish have increased number of iridophores than that of wild-type (WT) larvae indicating that *alx4a* and *gbx2* are sufficient to bias pigment cell fate towards iridophores (Fig. 4e). Considering that adult melanophores are mostly derived from melanophore stem cells after 2 dpf [45, 46], *alx4a* and *gbx2* might have minimal impact on embryonic melanophore development, and instead repress melanophore differentiation or migration in the adult melanophore stem cells.

### alx4a re-expression rescues iridophores

To rule out the possibility of off-target effects from CRISPR editing contributing to the observed phenotype, we verified that the ablation of iridophores was a direct consequence of *alx4a* loss through targeted rescuing with miniCoopR system. We generated multiple transgenic fish carrying *miniCoopR-alx4a* cassette with *alx4a*^*KO*^ background (Additional file 1: Fig. S11a). Two unique *alx4a* mutant alleles displayed clonal iridophore rescue and reestablishment of proper lateral stripe formation [40, 41, 47], suggesting that re-expression of *alx4a* during late NCC stage was sufficient to reestablish proper iridophore differentiation (Fig. 4f, Additional file 1: Fig. S11c). Interestingly, very faint to no GFP expression was detected in the rescued iridophores while the xanthophores strongly expressed GFP (Additional file 1: Fig. S11d-e). The detection of GFP in xanthophores indicates that the ectopic expression of *alx4a* does not impact xanthophore differentiation. In iridophores, the *mitfa* promoter loses transcriptional activity, which implies that the constitutive expression of *alx4a* is not necessary for cell maintenance or identity. This result could suggest that *alx4a* acts as a pioneer transcription factor to modify the epigenetic landscape but is dispensable afterwards.

## Discussion

In this study, we provide one of the first insights into the epigenetic dynamics that shape neural crest differentiation into pigment cells in zebrafish. We found that cell differentiation in zebrafish is characterized by promiscuous loss of DNA methylation coupled with dynamic chromatin accessibility. We report that the epigenetic features of DEG promoters are often static and that the majority of dynamic epigenetic changes occur in the intergenic or intronic regions. This suggests that the gene regulatory networks that define pigment cell fate are mostly regulated by enhancer-like cis-regulatory elements rather than promoter dynamics.

Here, we also present the previously uncharacterized epigenetic dynamics that define iridophore development. By pairing the epigenetic landscape with transcriptomes, we provide a putative gene regulatory network, potentially regulated by aristaless homeobox transcription factors, that is important for iridophore physiology. Although *alx4a* has been shown to be preferentially expressed in iridophores [27, 39], no mechanistical explanation or experimental validations on *alx4a*'s impact on iridophore differentiation has been provided. More importantly, genes preferentially expressed in certain cell types does not equate to importance to cell fate decision. For example, *alx1*, *alx3*, and *alx4b* are highly expressed in iridophore, but genetic ablation of these transcription factors did not impact iridophore differentiation in this study. However, the loss and restoration of *alx4a* expression resulted in a deficiency and recovery of body iridophores in zebrafish highlighting the important information that epigenetic-based analysis can provide in the study of cell fate determination. By examining the dynamic epigenetic landscape, we reveal that many genes in the guanine synthesis pathway have nearby latent cis-regulatory elements that might be regulated by *alx4a*. However, we acknowledge that further work is needed to verify that *alx4a* directly binds to these putative enhancers to induce nearby gene expression. Lastly, ectopic expression of *alx4a* and *gbx2* in early pigment progenitor cells leads to melanophore loss in adult transgenic fish. What roles *alx4a* and *gbx2* play in melanophore development and patterning is another intriguing question ripe for future studies.

## Conclusions

In conclusion, our work demonstrates that comparative epigenomics is a powerful tool that produces diverse hypotheses to dissect regulatory mechanisms that drive cell fate decisions. In this study, we produced DNA methylome and chromatin accessibility maps across pigment cell development and identified that pigment cell fate decisions are largely driven by enhancer activation rather than promoter dynamics. Furthermore, by profiling the epigenome dynamics, we identified numerous transcription factor candidates and cis-regulatory elements that might compel melanophore or iridophore cell fate. In this study, we performed genetic manipulations of *alx4a* and *gbx2* candidates to verify that both are necessary and sufficient for iridophore cell fate. Although one caveat of this study is the lack of mechanistic evidence that transcription factors bind to these putative enhancers, we hope that this study will be a valuable resource and welcomed addition to the impressive growing collection of epigenetic profiles [48] for the zebrafish community.

## Methods

### Zebrafish maintenance and strains

All fish procedures for this study were carried out following strict guidelines outlined in protocol #20140195, #20160109, and #20190041 approved by Washington University Animal Use Committee. The zebrafish strains utilized in this study were maintained according to standard conditions defined previously [49]. Neural crest cells were collected from *Tg(crestinA:EGFP)* line, in which 1200 bp of *crestin* element (*crestinA*) was cloned upstream of EGFP and integrated into the genome via Tol2 transgenesis [50]. Differentiated melanophores and iridophores were collected from *mlpha*^*j120*^ strain [51], a *melanophilin* mutant strain that displays reduced dispersion of melanosomes in melanophores. We chose *mlpha* ^*j120*^ to circumvent residual EGFP expression in *Tg(crestin:EGFP)* lines that might interfere with FACS isolation of pigment cells. For CRISPR and miniCoopR experiments, we utilized AB* strain for its availability.

### Neural crest cell and pigment cell isolation

*Tg(crestinA:EGFP)* labels neural crest cells (NCCs) from the 14–15 somite stage (neural crest formation) to differentiation into pigment cells. For 15-somite (early) and prim-5 (24 hpf late) neural crest cell isolation, *Tg(crestinA:EGFP)* embryos at designated biological time points were dechorionated with 20 mg/mL Pronase (Millipore Sigma, 10165921001), rinsed with egg water to remove the chorion, and collected into 1.5-ml Eppendorf tubes on ice. Then, 15-somite embryos were dissociated into single cells with deyolking buffer (55 mM NaCl, 1.8 mM KCl, and 1.25 mM NaHCO_3_) and gentle pipetting. A total of 24 hpf embryos were single-cell dissociated by adding Gibco TrypLE Express enzyme solution (ThermoFisher Scientific, 12604021) and incubating at 37 °C for 10 min followed by pipetting. To remove the dissociation buffer, single-cell dissociated samples were pelleted by centrifugation at 300*g* for 8 min at 4°C and the supernatant was discarded. The cell pellet was resuspended in 1× PBS + 2% FBS solution and filtered through 100-μm CellTrics filters (Sysmex-Partec, 04-004-2328). Samples were pelleted, resuspended, and kept on ice for subsequent FACS process. Then, 7-AAD dye (ThermoFisher Scientific, A1310) was added to sample 10 min prior to flow cytometry to label dead cells. Neural crest GFP-positive cells were sorted and collected on a Beckman Coulter MoFlo using a 70-μm nozzle.

For melanophore and iridophore isolation, we adapted a previously published protocol [27] developed by the Johnson lab. In brief, 4-5 dpf *mlpha*^*j120*^ larvae were anesthetized with Tricane for 15 min and collected into 50-ml conical tubes on ice. After removing the egg water, the larvae were digested with Gibco TrypLE Express enzyme solution in 37 °C shaking incubator (200 rpm) for 30 min. The larvae solution was filtered with a 120-μm filter to collect the dissociated cells. Melanophores and iridophores were isolated via Percoll (Millipore Sigma, P1644) density centrifugation. The purified pigment cell solution was further processed on a Beckman Coulter MoFlo (100 μm nozzle) to separate the melanophores and iridophores as detailed previously [27].

### Epigenome and transcriptome sequencing library construction

Genomic DNA (gDNA) for whole-genome bisulfite sequencing (WGBS) was purified from NCCs and pigment cells via phenol-chloroform:isoamyl alcohol (PCI) extraction and ethanol precipitation method. Then, 500 ng of gDNA was bisulfite treated using EZ DNA Methylation-Direct kit (Zymo, D5020) and processed with TruSeq DNA Methylation Kit (Illumina, 15066014) to generate Illumina-compatible WGBS libraries.

Chromatin accessibility maps were generated from 15,000–50,000 NCC and pigment cells by following a previously published ATAC-seq method [52].

We isolated total RNA via TRIzol Reagent (Thermo Fisher Scientific, 15596026) following the manufacturer’s recommendation. Then the total RNA was treated with TURBO DNase (Thermo Fisher Scientific, AM2238) to remove any residual DNA contamination. mRNA-seq libraries were then constructed with TruSeq RNA Library Prep Kit v2 (Illumina, RS-122-2001) following the manufacturer’s instructions. All libraries were sequenced on the Illumina NextSeq 500 platform (75 bp paired-end reads).

### Identification of DMRs

Paired-end reads from WGBS libraries were trimmed for adapter sequences with Cutadapt [53] and mapped to the danRer10 reference genome using Bismark [54] aligner with the following options: “-N 1 -L 28 –score_min L,0,-0.6.” Redundant aligned reads were identified and removed using Picard [55] MarkDuplicates command (http://broadinstitute.github.io/picard/). The bismark_methylation_extractor command from Bismark and a custom script were used to calculate DNA methylation levels for each CpG. Bisulfite conversion efficiency ranged from 98.5 to 98.9% (Additional file 2: Table S1).

To identify DMRs, biological replicates were combined to improve coverage of CpGs and then processed using the DSS tool [56] with standard parameters plus “smoothing = TRUE, delta = 0.30 (at least 30% methylation difference), and p.threshold = 0.01.” A DNA methylation Pearson correlation plot was generated using the “corrplot” package in R while other figures were generated using custom R scripts.

### Identification of DEGs and gene ontology enrichments

mRNA-seq libraries were adapter-trimmed and aligned to the danRer10 using STAR [57]. Gene transcript abundance (TPM) was calculated with StringTie [58] using Danio_rerio.GRCz10.85.gtf as a reference. We additionally processed aligned reads with HTSeq [59] to generate a gene count matrix, which was subsequently processed using DESeq2 [60] to identify differentially expressed genes. More specifically in DESeq2, we identified significantly differentially expressed genes by filtering for only genes with counts > 1, fold change > 2 and adjusted *p* value < 0.01. The DEG expression plot was generated using the Maplot function in DESeq2. Hierarchical clustering based on RNA expression was generated with the “pheatmap” package [61] in R.

To identify which gene ontologies are enriched in DEGs across NCCs and pigment cells, we further filtered the DEGs identified by DESeq2 for genes with TPM > 5 to remove lowly expressed genes. The list of DEGs was processed by Metascape [62] for GO term enrichment (*q* values for each GO term are presented in figures).

Since no comprehensive zebrafish transcription factor (TF) list was available at the time of analysis, we manually curated a zebrafish TF list with AnimalTFDB 2.0 [63]. Human TFs were converted into zebrafish orthologs using OrthoRetriever (http://lighthouse.ucsf.edu/orthoretriever/). Human TFs with no zebrafish orthologs detected by OrthoRetreiver were manually converted through literature search. Differentially expressed TF heatmaps were visualized using “ComplexHeatmap” package [64] in R.

### Identification of ATAC peaks and DARs

ATAC-seq reads were trimmed for adapter sequences and aligned to the danRer10 genome using bwa (bwa mem) [65]. Duplicate reads were removed with Picard MarkDuplicates. Then the libraries were downsampled to 35 million aligned reads to minimize artifacts introduced by library size difference for peak calling analysis. Since the ends of the reads represent Tn5 insertion locations, we processed the aligned reads by offsetting + strand reads by + 4 bp and – strand reads by − 5 bp. The offset position for each read was used as input for calling peaks with MACS2 [66] using the following parameters: “-g 1.4e9 -B –SPMR –keep-dup all –nomodel -s 75 –extsize 73 –shift -37 -p 0.01”. With narrowPeak output from MACS2, we utilized irreproducible discover rate (IDR) framework [67] to generate a consensus peak file from each biological time point. To identify differentially accessible regions, we processed ATAC peaks with DiffBind [68] with a stringent cutoff of FDR < 0.001.

To characterize super-accessible regions, we adapted the data analysis method for superenhancer detection [33]. Iridophore-specific opening DAR peaks within 12.5 kb were merged together using the BEDTools [69] merge command, and the cutting frequencies (tagmentation events marked by start and end of paired reads) were calculated for each peak. Then the peaks were then ordered from least to most tagmentation events to identify the cutoff for super-accessible region detection (Additional file 1: Fig. S2e). The closest gene promoters to these super-accessible regions were identified using BEDTools closest command.

### Identification and characterization of DMARs

Differentially methylated and/or accessible regions were classified by identifying overlapping DMRs and DARs with the BEDTools [69] intersect command (Additional file 2: Table S2). DMARs were annotated for their genomic location using HOMER [70] annotatePeaks.pl. Furthermore, we performed BEDTools intersect to detect DMARs located within 50 kb of DEG promoters. We calculated average phastCons and phyloP conservation scores by liftOver of danRer7 phastCons vertebrate 8-way [34] and phyloP vertebrate 8-way [35] scores to danRer10 [71]. Shuffled genomic regions were generated by using BEDTools shuffle on melanophore-specific DARs or iridophore-specific DARs.

For gene ontology enrichment analysis on DMRs, DARs, and DMARs, we identified the closest differentially expressed gene promoter to these epigenetically dynamic regions using the BEDTools closest command. For example, for shared hypoDMRs, we identified melanophore-specific closest DEG promoters and also performed separate identification for iridophore-specific closest DEG promoters for downstream gene ontology enrichment analysis. We analyzed this list of genes with Metascape [62] for GO term enrichment (*q* values for each term are presented in figures).

All DMRs, DARs, and DMARs were processed with AME [72] from MEME suite using JASPAR_CORE_vertebrates_non-redundant_PFMs [73] to discover which known motifs are enriched in these epigenetically dynamic regions. Motif footprint signatures in DMARs were generated by CENTIPEDE [74]. For each DM/AR, we used FIMO [75] to scan and detect the presence of particular motifs. Line graphs and heatmaps representing DNA methylation levels and chromatin accessibility across DM/ARs were generated by deepTools [76].

### Generation of putative iridophore-specific transcription factor network

Using motif enrichment results from iridophore-specific DMRs, DARs, and DMARs, we curated a list of previously known transcription factors related to iridophore development (*sox10* [10], *foxd3* [9, 22], *tfec* [23], and *gbx2* [24]*).* In murine model, *Ets1* has been shown to be essential for *Sox10* expression for proper melanocyte development [77]. Since *ets-1* was found to be enriched in iridophore DMARs, we decided to incorporate *ets-1* and *alx* TF candidates into the putative transcription factor network. Using transcriptomic data, we ordered the TFs based on the presence and abundance of TF expression across NCC differentiation into iridophores. Then we queried all DM/ARs within 50 kb of TF promoters for presence of various TF motifs using FIMO and created putative TF-TF connections to create the network.

### CRISPR-mediated knockout of *alx* transcription factors in zebrafish

To design gRNA sequences, we took advantage of CRISPOR [78] and CRISPRscan [79] algorithms to maximize specificity (CRISPOR) and efficacy (CRISPRscan). For each gRNA, a primer was ordered with the chosen gRNA sequence preceded by “5‘-aattaatacgactcactata-3‘” and followed by “5‘-gttttagagctagaaatagc-3‘.” Each gRNA primer was then annealed to the universal primer scaffold, “5‘-ttttgcaccgactcggtgccactttttcaagttgataacggactagccttattttaacttgctatttctagctctaaaac-3‘”. The sgRNAs were then transcribed in vitro using T7 RNA polymerase from the HiScribe™ T7 Quick High Yield RNA Synthesis Kit (New England Biolabs, E2050S). Cas9 mRNA was generated via in vitro RNA transcription of the pCS2-nls-zCas9-nls plasmid (Addgene, 47929) with mMessage mMachine SP6 Transcription Kit (Thermo Fisher Scientific, AM1340).

For each target gene, a 5-μl injection cocktail was made with 2 μg of zCas9 mRNA, 0.5 μl of 1% or 0.5% phenol red dye, 400 ng of each of the two sgRNAs targeting a gene of interest. 0.5 nL of the CRISPR cocktail was injected directly into the cell of single-cell embryo (AB*). To identify founders with indels in target genes, we pairwise crossed CRISPR-injected adult fish and collected embryos to PCR amplify target gene locus and performed a T7 endonuclease I (NEB, M0302S) assay on the amplified region. All homozygous indels were verified via Sanger sequencing.

### Ectopic expression of *alx4a* and *gbx2* with miniCoopR vector in wild-type AB* and *alx4a* mutant fish

To ectopically express transcription factors in pigment progenitor cells, we exploited the miniCoopR system [44, 80]. We generated *alx4a* and *gbx2* CDS fragments from PCR amplifying cDNA from reverse transcribed mRNA extracted from 24 hpf AB* (WT) embryos. Since early pigment progenitor cells express *mitfa*, we cloned in candidate CDS in lieu of *mitfa* minigene via Gibson Assembly. We injected approximately 1 nl of 100 ng/μl miniCoopR vector and 15 ng/μl Tol2 capped transposase mRNA cocktail into the yolk of single-cell embryos. All GFP+ F0 embryos were raised to adulthood and screened for founders. Founders from AB* transgenic fish were crossed to generate F1 transgenic fish. Adult F1 miniCoopR transgenic fish were screened for pigment phenotype and bred to generate 3 dpf larvae for iridophore quantification in tail trunks. For the rescue experiment, GFP-positive F0 adults were screened for iridophore recovery. GFP, RFP, and white light pictures were taken using a Nikon SMZ18 fluorescent microscope with NIS-Elements AR (4.30.02) software.

## Supplementary Information

**Additional file 1: **Supplemental Table Legends and **Figures S1-S11**

**Additional file 2: Table S1-S8**

**Additional file 3:.** Review history

## Data Availability

All sequencing data presented in this paper has been deposited and publicly available through Gene Expression Omnibus (GEO) repository: GSE181287 [81]. The code used for the analysis in this study is available on Zenodo (10.5281/zenodo.5496254) [82] and Github (https://github.com/joshhjang/zebrafish_pigment_cell_dev) [83] under the MIT license.
