# Supplementary material for: Epigenetic dynamics shaping melanophore and iridophore cell fate in zebrafish
Source: Genome Biol. 2021 Oct 4;22:282. doi: 10.1186/s13059-021-02493-x (PMC8489059; doi:10.1186/s13059-021-02493-x)
Supplement: Supplementary file 1 — Additional file 1: Supplemental Table Legends and Figures S1-S11 [file 13059_2021_2493_MOESM1_ESM.docx]

**Supplemental Tables:**

**Table S1: WGBS alignment conversion and CpG coverage metrics.** Table presenting the number of non-redundant uniquely mappable reads for each WGBS library. The lambda conversion rate and average CpG coverage is also presented.

**Table S2: DMR, DAR, DMAR location and annotation list.** Table presenting the detailed annotations (DMR, average methylation, DAR, IDR peak detection, CpG count, average CpG coverage and density) of all DMARs presented in this study.

**Table S3: TF motif enrichment in shared hypoDMR, openingDAR, and hypo-openingDMAR.** Table presenting the TFs that are differentially expressed and have motifs enriched in shared hypoDMR, openingDAR, and hypo-openingDMAR**.**

**Table S4: TF motif enrichment in shared closingDARs.** Table presenting the TFs that are differentially expressed and have motifs enriched in shared closingDARs**.**

**Table S5: TF motif enrichment in melanophore-biased closingDARs.** Table presenting the TFs that are differentially expressed and have motifs enriched in melanophore-biased closingDARs**.**

**Table S6: TF motif enrichment in iridophore-biased closingDARs.** Table presenting the TFs that are differentially expressed and have motifs enriched in iridophore-biased closingDARs**.**

**Table S7: Motif presence in DM/ARs within 50kb of iridophore DEGs.** Table presenting the number of motif occurrences in DM/ARs within 50kb of iridophore DEGs from top iridophore-enriched GO terms.

**Table S8: Primer sequences.** Primer sequences used to generate CRISPR gRNAs and miniCoopR vectors.

**Supplemental Table 8: Primer sequences.** Primer sequences used to generate CRISPR gRNAs and miniCoopR vectors.


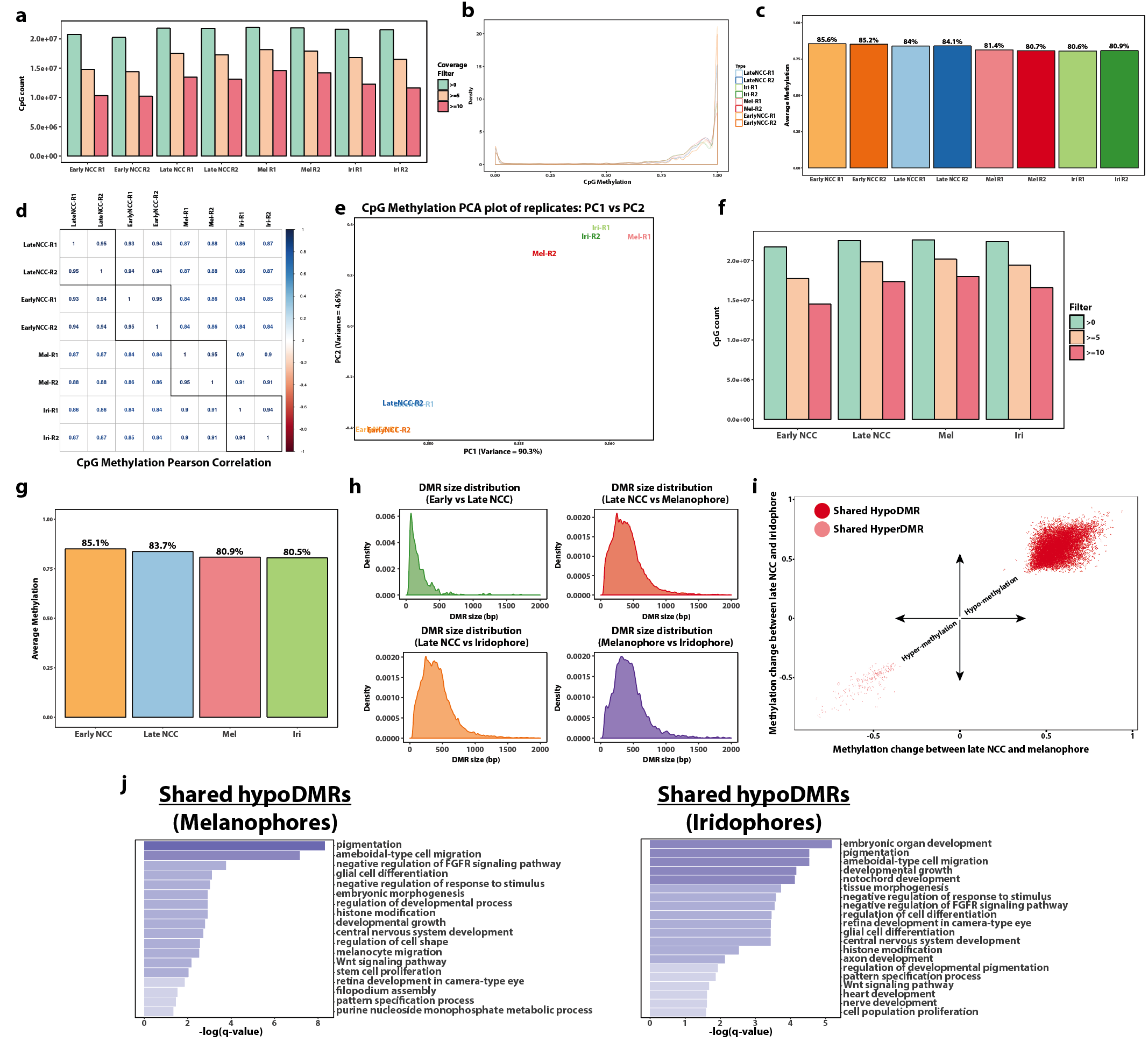
 **Fig S1: WGBS metrics and DNA methylation landscape from NCC and pigment cells.** **a,** Bar graph illustrating the number of CpGs captured at certain coverage cutoff for each biological replicate. **b,** Distribution of CpG methylation of each biological replicate. **c,** Average global methylation levels for each biological replicate. **d,** Pearson correlation of CpG methylation across biological replicates. **e,** Principal component analysis on CpG methylation of biological sample. PC1 and PC2 variance is presented. **f,** Bar graph illustrating the number of CpGs captured at certain coverage cutoff for combined biological replicate. **g,** Average global methylation levels for combined biological replicate. **h,** Distribution of DMR sizes for each pairwise comparison **i,** Scatter plot comparing methylation changes from shared DMRs between late NCC-melanophore and late NCC-iridophore. **j,** Gene ontology enrichment of DEGs closest (within 50kb) to shared hypoDMRs from late NCC to pigment cell comparisons.

**
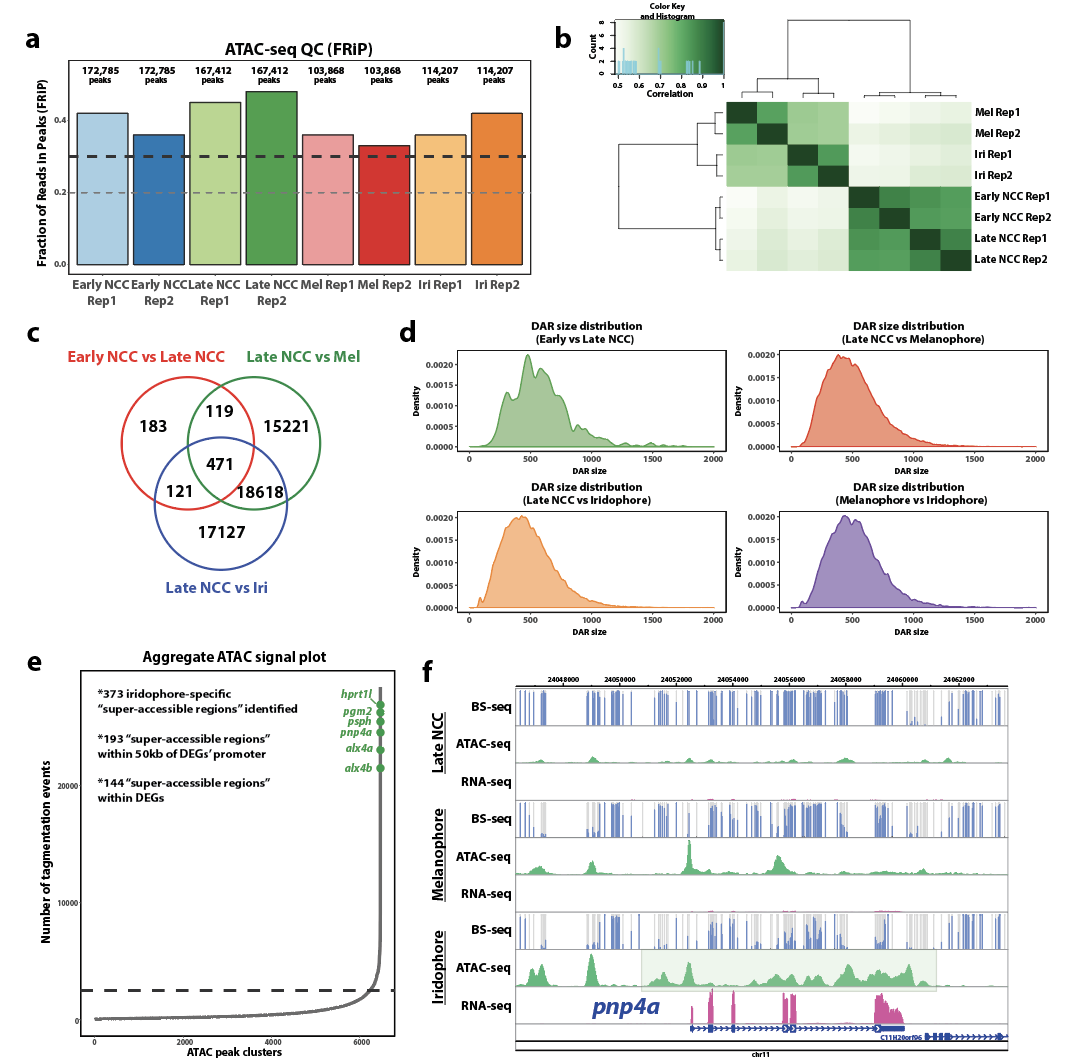
 Fig S2: Chromatin accessibility landscape in NCC and pigment cells.** **a,** Bar plot illustrating fraction of reads in peaks (FRiP) for each biological sample. ENCODE standard for passable and high quality is marked by grey and black dashed lines, respectively. **b,** Correlation heatmap of ATAC-seq peaks across biological replicates. **c,** Venn diagram of overlapping DARs for each comparison. **d,** Distribution of DAR sizes for each comparison. **e,** Tagmentation frequency aggregate plot to identify most accessible peaks in iridophores. Green dots represent top iridophore-specific DEGs that have super-accessible regions within 50kb of promoter. **f,** WashU Epigenome browser view of *pnp4a* gene. Example of super-accessible region is highlighted by the green box in iridophore ATAC-seq track.

**
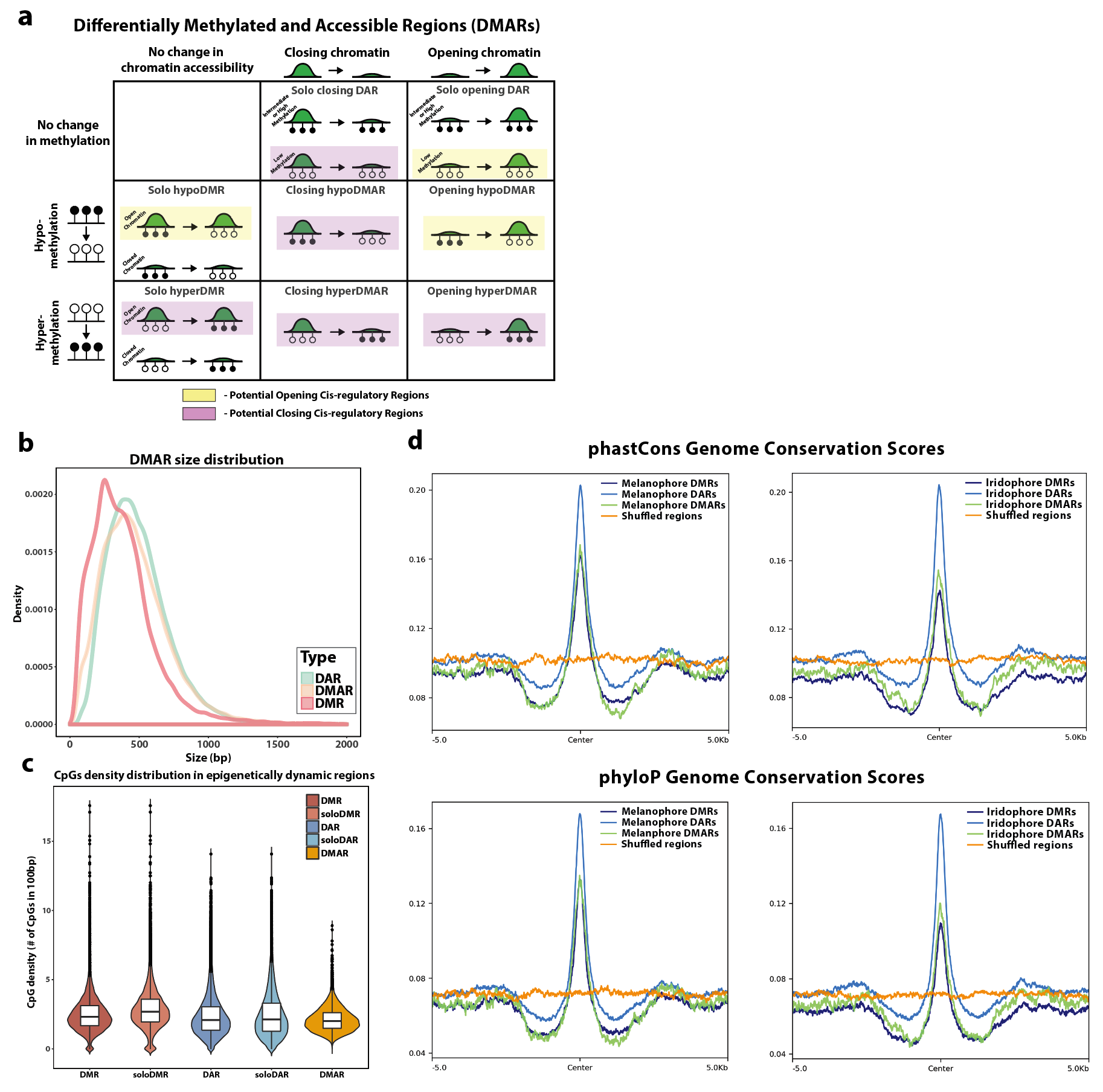
**

**Fig S3: DMAR characteristics.** **a,** Schematic describing various combinations of methylation and chromatin accessibility states of a genomic region. **b,** Distribution of DMR, DAR, and DMAR sizes. **c,** Violin and box plot of CpG density in various epigenetically dynamic regions. **d,** Average vertebrate phastCons and phyloP scores of melanophore-specific or iridophore-specific DMRs, DARs, DMARs, and sequence-shuffled regions and their flanking 5 kb regions are plotted.

**
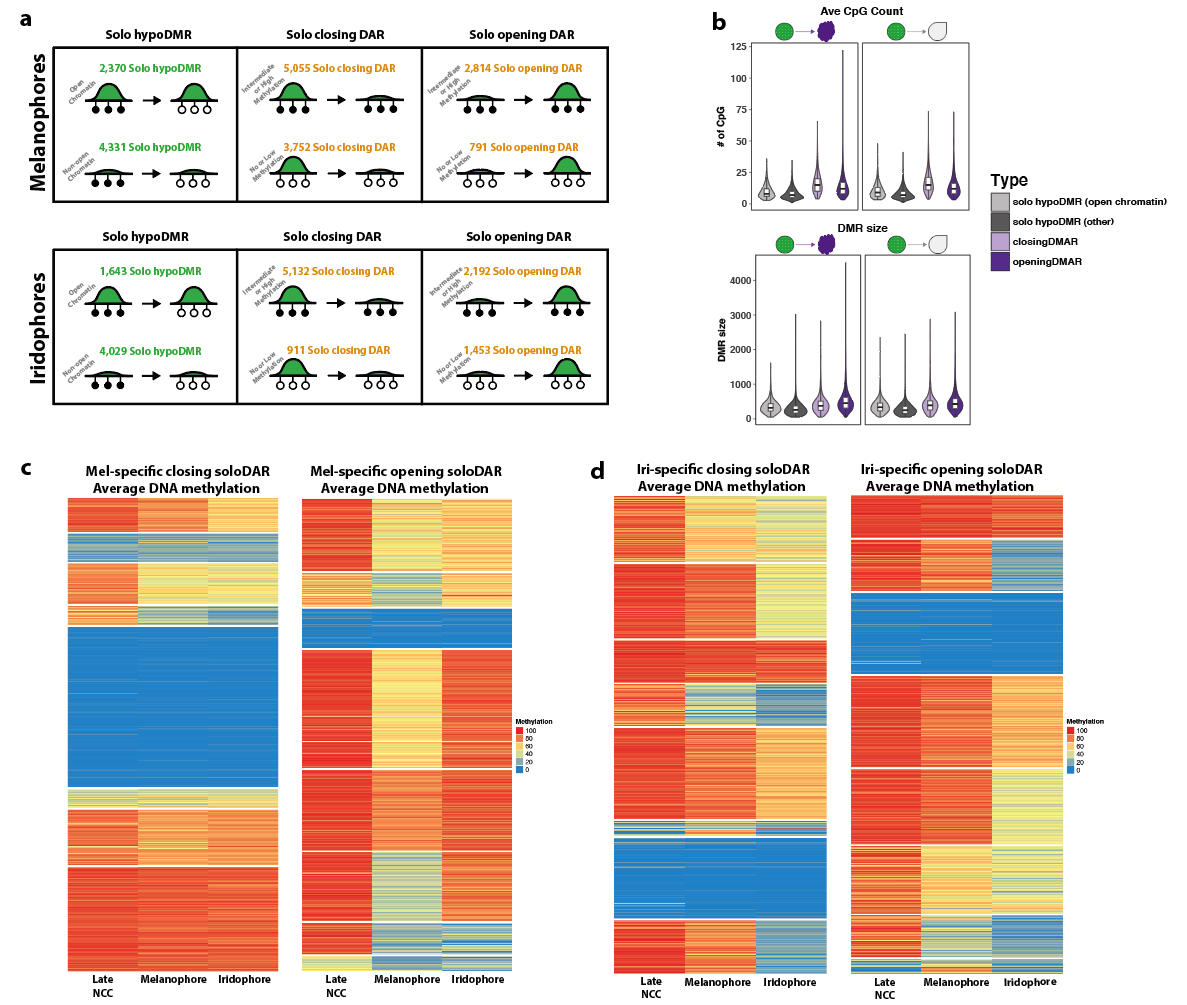
**

**Fig S4: Detailed characterization of solo DMRs and solo DARs**. **a,** Schematic and quantification of pre-existing epigenetic states in solo hypoDMRs, solo closingDARs, and solo openingDARs in melanophores and iridophores. In solo hypoDMRs, open chromatin is specified if ATAC peak is detected after IDR filter. For dynamic DARs, no or low DNA methylation demarcates 0-30% methylation while intermediate or high methylation represents > 30% DNA methylation levels. **b,** Violin plot illustrating the distribution of CpG count and DMR/DMAR size of solo hypoDMRs and DMARs. **c-d,** Heatmap illustrating methylation levels of melanophore-specific solo DARs (**c**) and iridophore-specific solo DARs (**d**) in late NCC, melanophores and iridophores. Eight clusters of methylation patterns generated by k-means clustering are presented.


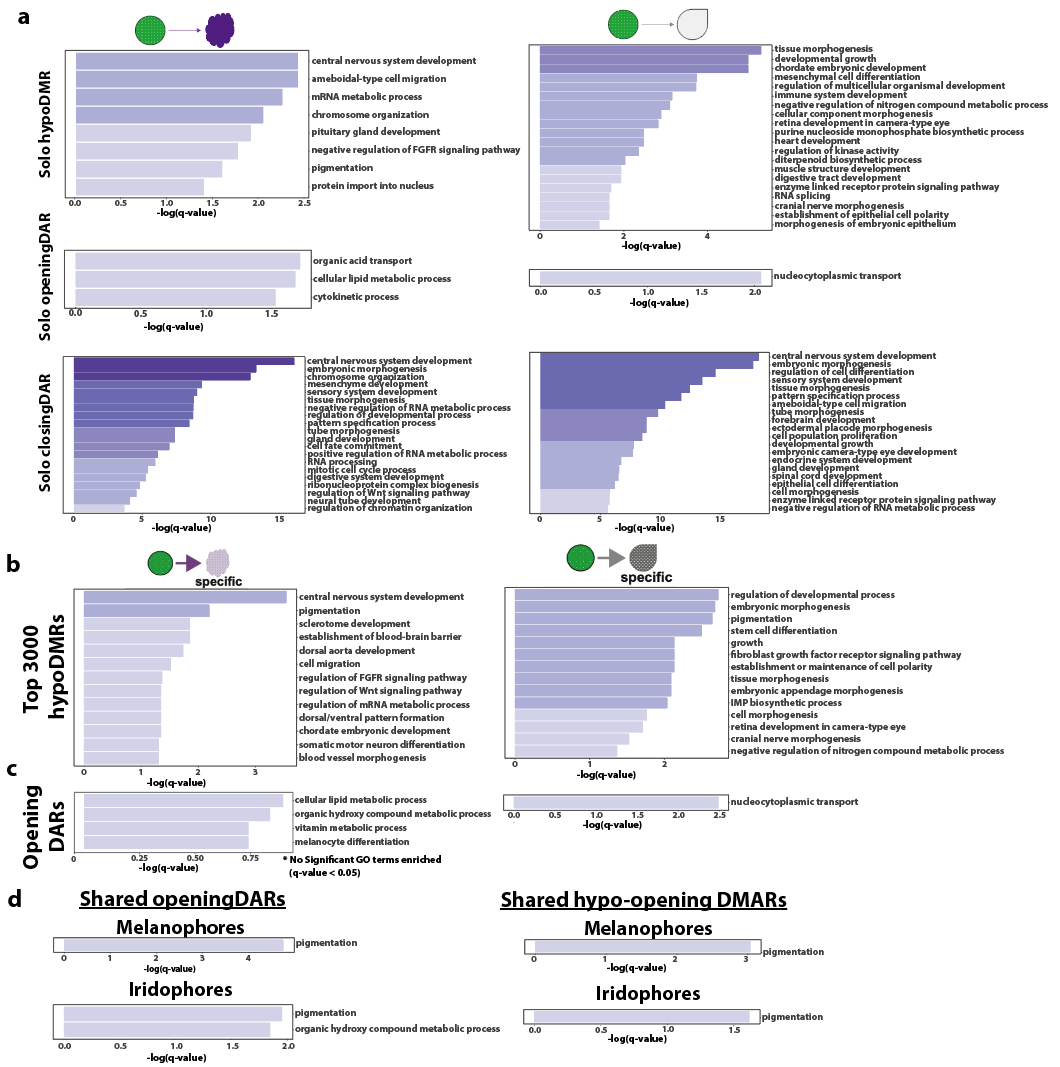


**Fig S5: Gene ontology enrichment analysis of epigenetically dynamic regions. a,** Gene ontology enrichment of DEGs closest (within 50kb) to solo hypoDMRs, solo openingDARs, and solo closingDARs from late NCC to pigment cell type comparison. **b-c,** Gene ontology enrichment of DEGs closest (within 50kb) to pigment cell-specific top 3000 hypoDMRs (**b**) and opening DARs (**c**). **d,** Gene ontology enrichment of DEGs closest (within 50kb) to shared opening DARs and hypo-opening DMARs between melanophores and iridophores.

**
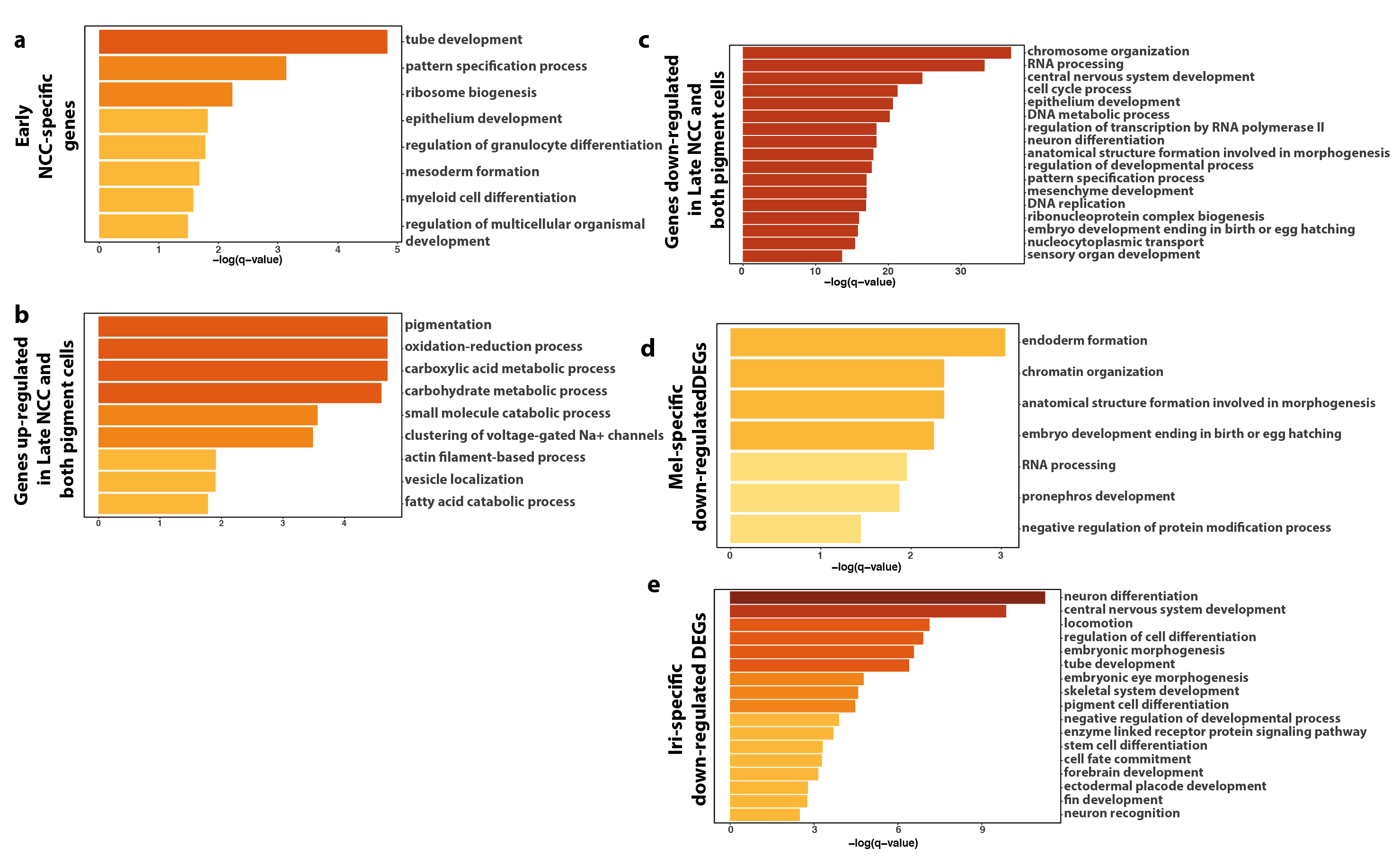
**

**Fig S6: Gene ontology enrichment of various DEG categories.** **a,** GO terms enriched in genes expressed in early NCC and down-regulated in late NCC. **b,** GO enrichment of genes up-regulated in late NCC, melanophores, and iridophores. **c,** GO enrichment of genes down-regulated in late NCC, melanophores, and iridophores. **d,** GO enrichment of melanophore-specific down-regulated genes. **e,** GO enrichment of iridophore-specific down-regulated genes.


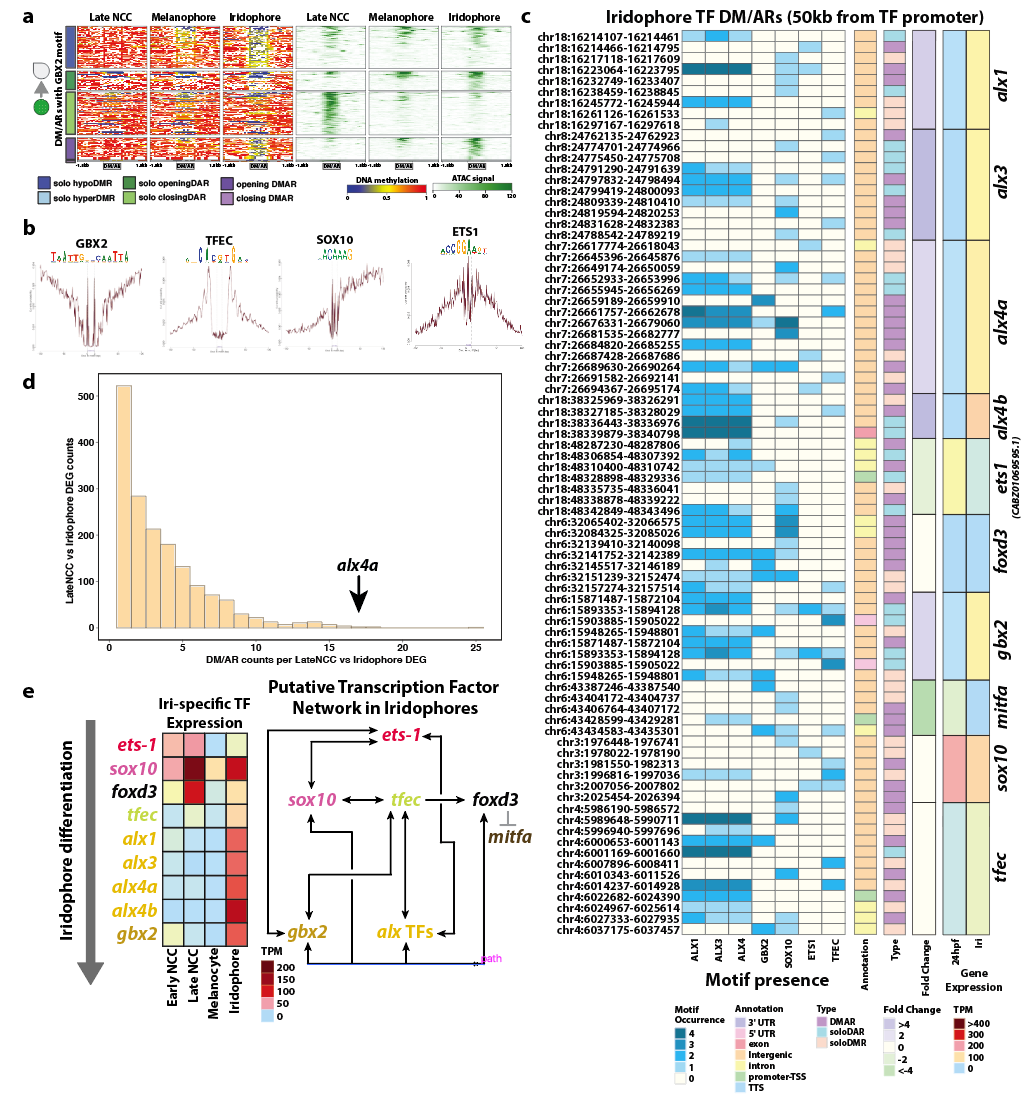


**Fig S7: Characterization of motif presence in epigenetically dynamic regions within 50kb of DEG promoters.** **a,** Heatmaps representing DNA methylation and ATAC signal across iridophore-associated DM/ARs with GBX2 motifs. **b,** ATAC-seq footprint signatures of additional iridophore-specific transcription factor candidates. **c,** Heatmap portraying number of motif instances of ALX1, ALX3, ALX4, GBX2, ETS1, SOX10, and TFEC, and genomic annotation, type of epigenetic dynamics, gene fold change from late NCC to iridophores, and gene expression levels of various transcription factors. **d,** Histogram displaying the frequency of DM/ARs within 50kb of Late NCC vs Iridophore up-regulated DEGs' promoters. **e,** Heatmap representing transcription factor gene expression in earlyNCC, lateNCC, melanophores and iridophores and a putative transcription factor network for iridophore differentiation.

**
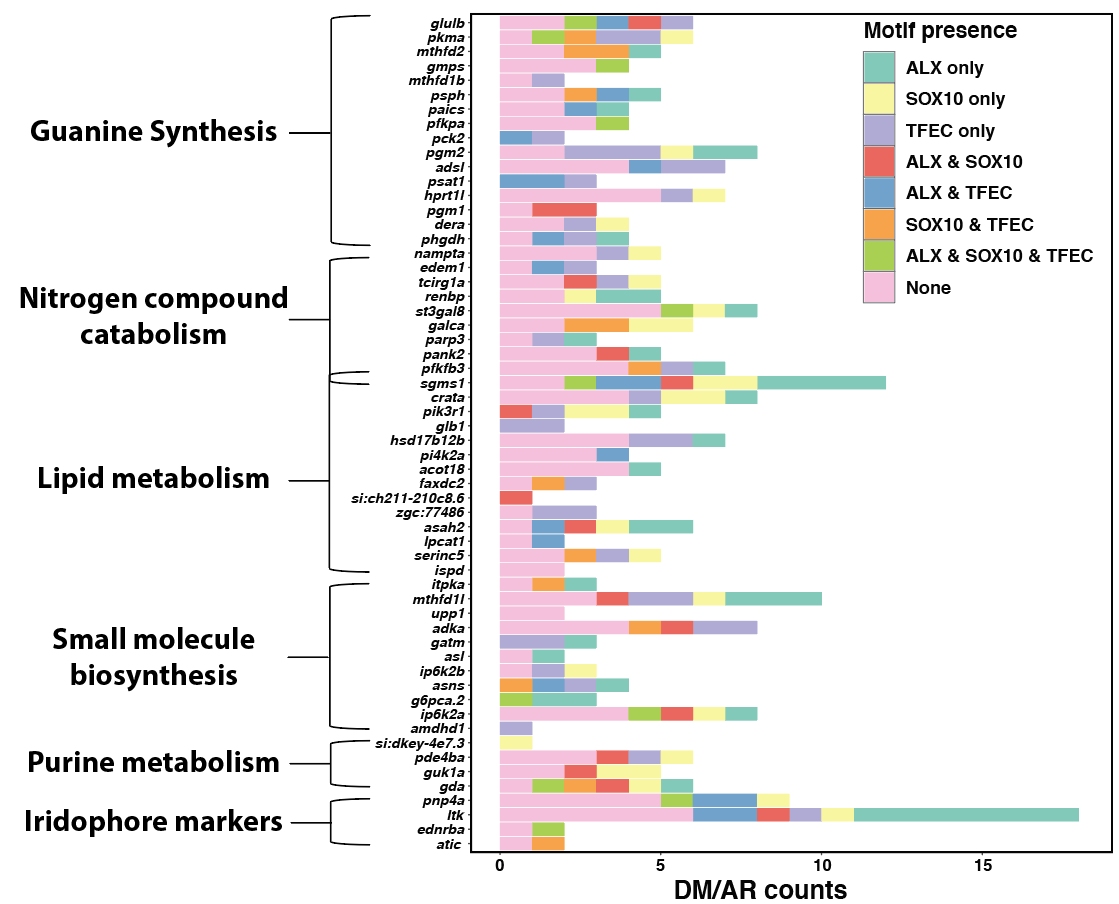
**

**Fig S8: Characterization of motif presence in epigenetically dynamic regions within 50kb of DEG promoters from enriched GO groups**. Frequency of DM/ARs with transcription factor motifs within 50 kb of iridophore-specific up-regulated gene promoters across various iridophore-enriched GO groups.

**
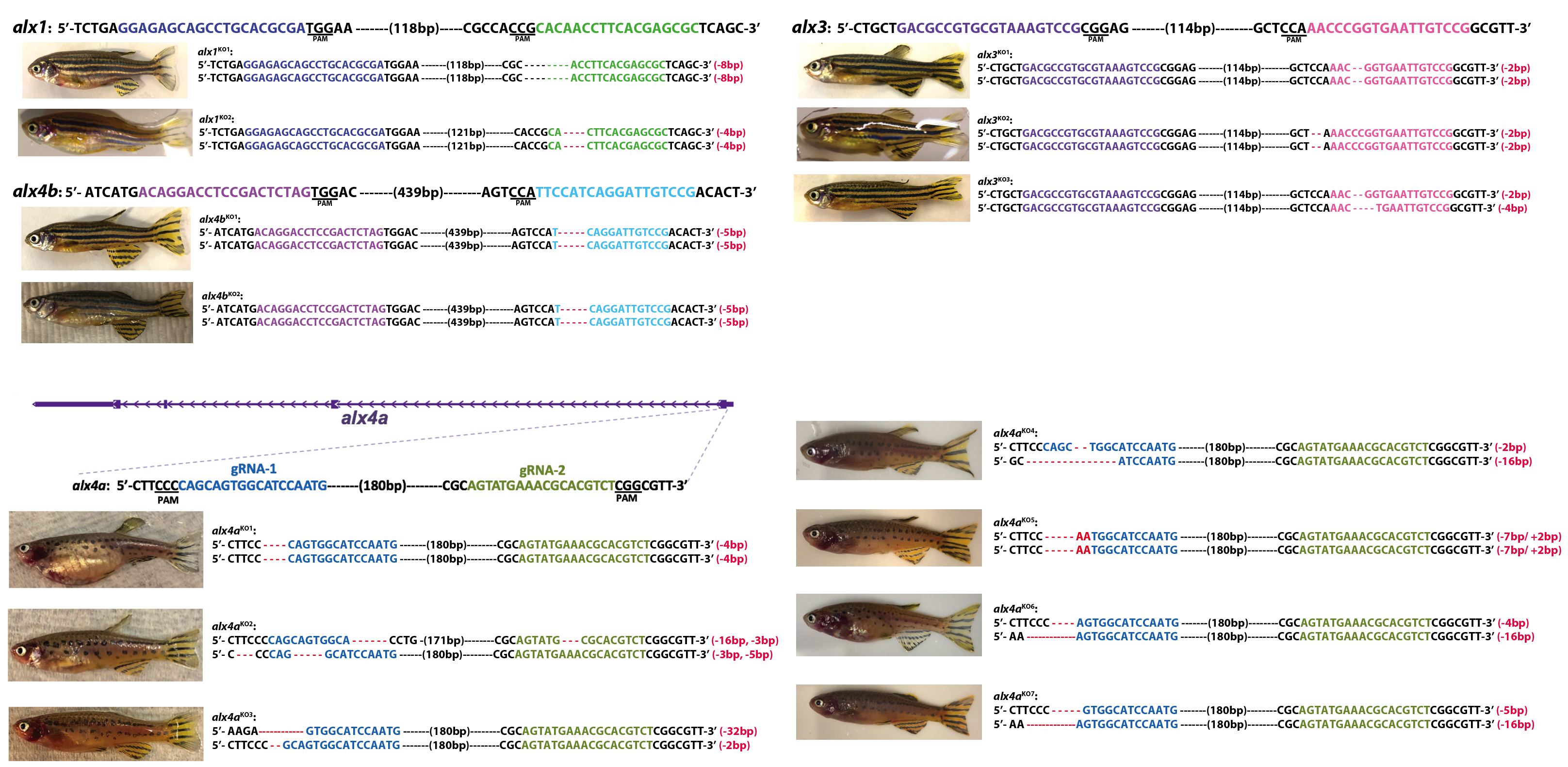
**

**Fig S9: CRISPR-mediated mutant genotypes**. Sequence of wild-type allele with gRNA sequence (colored) and PAM motif (underlined) designated. Lateral whole-body pictures of *alx1, alx3,* and *alx4b* mutant fish coupled with frameshift indel genotype. Below is a schematic illustration of *alx4a* CRISPR target location and sequence. Lateral view of *alx4a* knockout fish with unique frameshift alleles are presented.

**
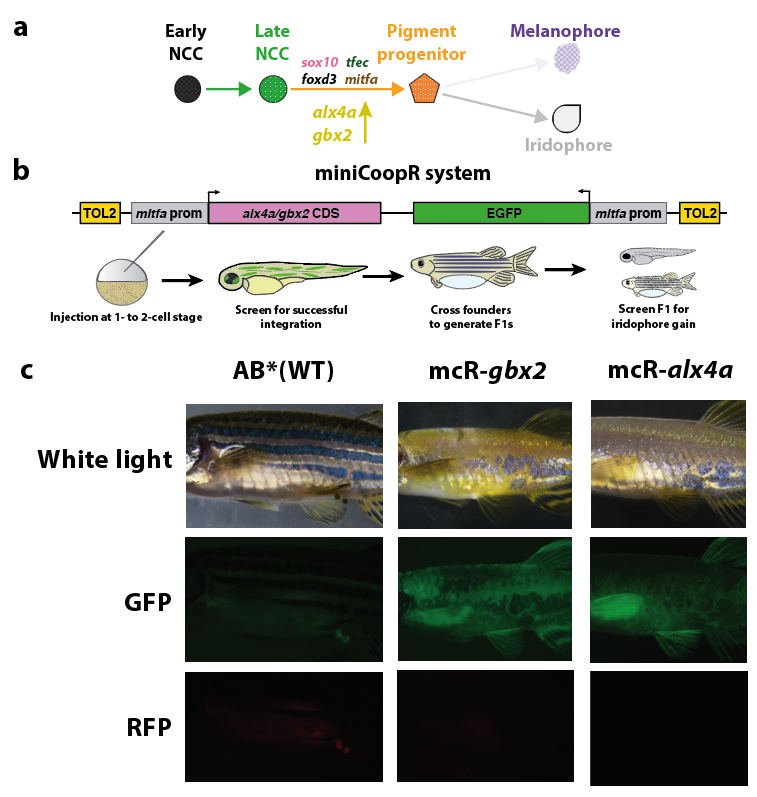
**

**Fig S10: Magnified view of *Tg(miniCoopR-alx4a)* and *Tg(miniCoopR-gbx2)* fish.** **a,** Schematic illustrating the timepoint when *alx4a* and *gbx2* will be ectopically expressed behind *mitfa* promoter during embryogenesis. **b,** Schematic describing the minicoopR-*alx4a* and miniCoopR-*gbx2* vectors and experimental design. **c,** GFP expression in transgenic fish indicates successful integration and expression of miniCoopR vector in wild-type AB* background. The GFP expression is not due to autofluorescence as indicated by lack of RFP detection.

**
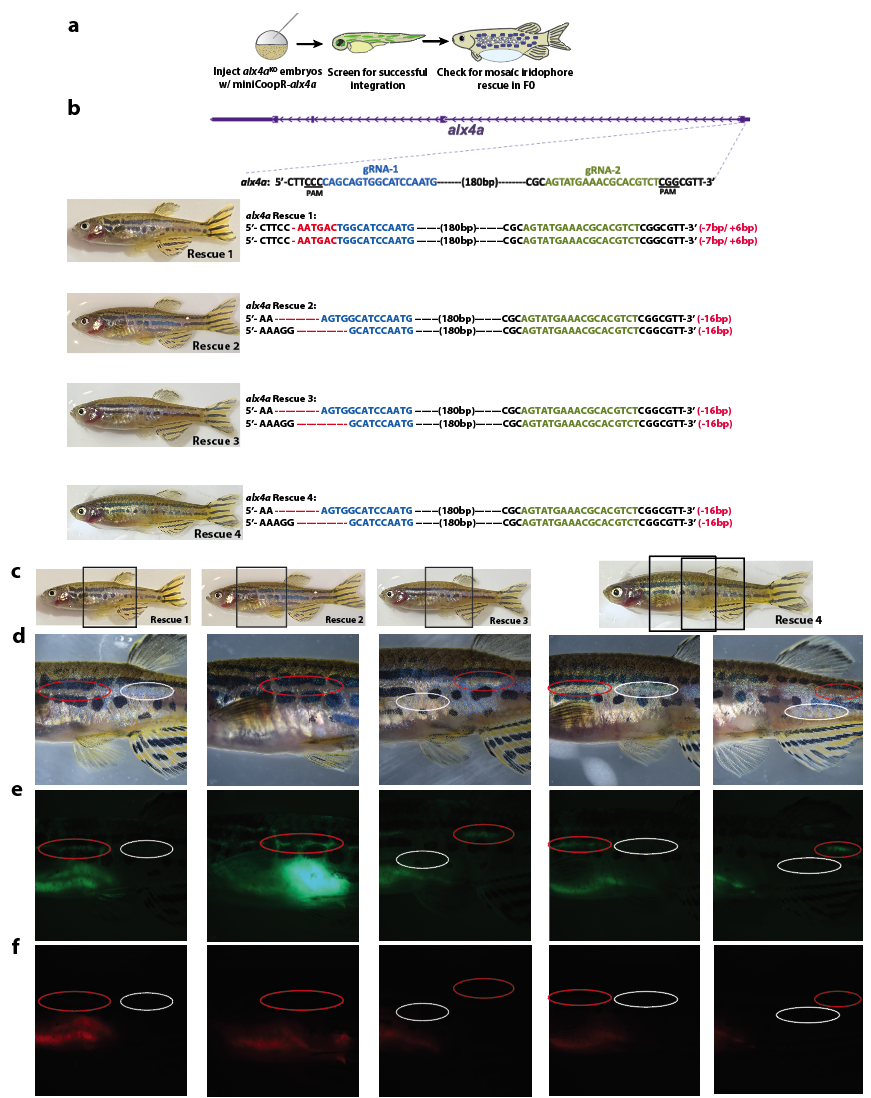
**

**Fig S11: miniCoopR-*alx4a;alx4a*-KO transgenic fish.** **a,** Schematic describing experiment design using minicoopR-*alx4a* vector to re-express *alx4a* in *alx4a*-KO fish. **b,** Schematic illustration of *alx4a* CRISPR target location and sequence. Lateral view of four miniCoopR-*alx4a;alx4a* rescue fish with frameshift alleles in *alx4a* first exon are presented. **c-d**, Black box **(c)** denotes the zoomed in region depicted in picture below (**d**)**. e,** GFP detection in zoomed regions. Red circle and white circle represent GFP-positive xanthophores and GFP-negative iridophores in both regions. **f,** RFP detection in zoomed regions.
