# Supplementary material for: Epigenetic dynamics shaping melanophore and iridophore cell fate in zebrafish
Source: Genome Biol. 2021 Oct 4;22:282. doi: 10.1186/s13059-021-02493-x (PMC8489059; doi:10.1186/s13059-021-02493-x)
Supplement: Supplementary file 3 — Additional file 3:. Review history [file 13059_2021_2493_MOESM3_ESM.docx]

Review History

**First round of review**

**Reviewer 1**

**Are you able to assess all statistics in the manuscript, including the appropriateness of statistical tests used?**

No, I do not feel adequately qualified to assess the statistics.

**Comments to author:**

The manuscript by Jang et al. presents a substantial amount of meticulously collected and analyzed data about the epigenetic progression of pigment cell differentiation in zebrafish. Starting with 4 purified cell populations, they track changes in gene expression, methylation, and chromatin accessibility through the early stages of neural crest cell commitment and the formation of two pigment cell types, melanophores and iridiphores, that form from a common pigment precursor cell. They present the analyses clearly, and many of their findings confirm known aspects of NCC and pigment cell differentiation. Importantly, they also reach a number of significant and novel conclusions. First, they look specifically at the overlap of differentially methylated regions (DMRs) and differentially accessible regions (DARs), so-called DMARs. These are not only better correlated with nearby differentially expressed genes (DEGs) but also tend to correspond to evolutionarily conserved regions. Although they emphasize the finding in the context of iridiphores, it is likely generalizable to many cell differentiation processes. Second, they find that the promoters of DEGs do not show correlative changes in either methylation or accessibility, but instead the correlative DMARs are located in intergenic and intronic regions, suggesting that the gene expression changes driving differentiation are regulated through distal enhancers, not promoters. Finally, they use motif analysis of the DMARs to identify potential regulatory transcription factors, and implicate the alx gene family. They confirm the functional significance by generating mutants of all alx genes, showing that alx4a mutants specifically lack iridiphores. Furthermore, ectopic expression of alx4a, and the previously identified iridiphore gene gbx2, prevent melanocyte formation, presumably by driving development of the common pigment precursor to the iridiphore lineage.

The data and the analysis are thorough and high quality. The conclusions are well-supported by the data. If anything, the authors have undersold their work. I have only a few minor comments:

1. In the title and throughout, I would argue that melanophore is the correct term, not melanocyte.

2. On the title page, the superscript indicates that Ting Wang is deceased (!), which I am certain must be a mistake, since he is the corresponding author.

3. There are several typos in the reference list. Most are minor and would be caught in final editing, but #14 did not have enough information to make it possible to find the paper.

4. In Supp Fig 3a, the colors in the overlapping peaks are very hard to see - perhaps patterns would work better, or just showing the outline of the curves instead of filling them in?

**Reviewer 2**

**Are you able to assess all statistics in the manuscript, including the appropriateness of statistical tests used?**

Yes, and I have assessed the statistics in my report.

**Comments to author:**

Authors of Jang et al. state that the epigenetic landscape contributing to pigment cell differentiation is poorly understood. To investigate this, they charted the global changes in the epigenetic landscapes, DNA methylation and chromatin accessibility, during neural crest differentiation into melanocytes and iridophores, with the goal to identify epigenetic determinants. They use crestin:GFP as a marker to separate NCC stages and then did WGBS-Seq, RNA-Seq, and ATAC-Seq. This leads them to ALX4, and they then perform experimental studies to test ALX4 function in zebrafish.

This is a well written manuscript with significant results. The experiments presented in figures 3 and 4 are quite interesting and appear conclusive. While I find the overall story compelling, I have major issues with the initial epigenetics studies which led to their investigation of ALX4. These issues, mostly concerning figure 2, cause the manuscript to be very disjointed and confusing.

Issues:

WGBS-Seq data as presented in figures is near impossible to interpret - Fig 1b specifically. Please separate the panel with CpG sites so that methylation levels can be seen clearly. Statement "associated with low promoter methylation and high chromatin accessibility" is not supported. This statement requires a statistical test comparing these regions, and I do not see this test in the manuscript.

For DMR calling, biological replicates were combined to improve coverage of CpGs, but this approach may lead to increased false positive DMRs. Authors should evaluate identified DMRs measuring methylation for individual replicates to determine whether DMRs might be caused either by low read coverage or high methylation variability. If necessary, further filtering of DMRs should be applied to eliminate false positives.

At line 113 - what is meant by "these shared DMRS could predict the DNA methylation landscapre of the intermediate pigment progenitor cell"?... do they mean that DMRs have this potential, or can DMRs were actually do this, because I find nowhere in the manuscript that authors attempted to make unsupervised predictions based on methylation status?

Authors indicate they focused on "dynamic" DMARs, but how do they define "dynamic" and in what manner did they focus on these regions? In figure S4 they begin analysis of what might be dynamics regions, comparing methylation levels at different stages in open or closed regions, but there is no downstream analysis. Analysis in figure S5 is not sufficient. What type of biologically relevant information can be gleaned from separate types/clusters of "dynamic" DMAR regions?

The general and somewhat broad characterization of differentially accessible and differentially methylated regions in figure 2 seems disjointed and unfocused. I would have much preferred more focused analysis investigating the overlap of DARs and DMRs, including in depth characterization of regions that meet the general expectation - regions becoming open also lose DNA methylation and closed regions become methylated - but authors fail to investigate regions to do not meet this expectation. For example, authors indicate that 4000/6000 iridophore-specific solo hypoDMRs occur at closed regions, but they do not investigate these regions further. If the genome is generally becoming more demethylated as late NCCs differentiate, then why are most regions less accessible? I would have expected the opposite. They make note of this issue in lines 138-140, but they do not investigate it more deeply.

At line 157, authors performed GO analysis on DMARs and state that "For both melanocyte and iridophore DMARs, numerous essential neural crest... were enriched" It seems in figure 2E that the most enriched pathways are for general developmental pathways, and not NCC-specific pathways. Authors should adjust their wording to correct this issue. They do identify pigment cell and neural crest cell related GO terms when they performed GO analysis based on gene expression levels, but the question remains - how do accessibility changes and DNA methylation changes relate the expression changes?

These issues with initial characterization or epigenetics data make the transition to figure 3 quite difficult and abrupt. The remaining portion of the paper rests completely on their identification of an enriched motif. I would have been nice if the authors measured epigenetic changes (DNA methylation and accessibility) relative ALX4 motifs across the genome. This would help the reader understand how general and dynamic ALX4 binding might be during iridophore differentiation. Once they've identified ALX4, the paper moves in a completely different and interesting direction, almost making the first half of the paper seem irrelevant. More in-depth analysis of epigenetic changes relative to ALX4 would help the reader understand how epigenetic factors may influence ALX4, and also help to connect the first half of the paper with the second half.

More details are needed to understand how authors generated "putative transcription factor network that drive iridophore cell fate" notes in line 184. Generally, this network seems somewhat interesting, but has little impact on the results of the manuscript. I suggest putting less emphasis on this result. Authors highlight this network in the abstract, and it sets the expectation that a bioinformatically established and experimentally validated network was constructed. These things were not done, and so I recommend eliminating this claim.

Indeed, the re-expression of ALX4 rescues iridophore differentiation in mutant fish, but re-expression also seems to rescue the uncharacterized melanocyte phenotype - why do the broken lateral stripes in mutants become more normal continuous stripes in the ALX4 over-expression fish?

Authors state that they would like to map ALX4 localization in the genome, and they cannot do this until an antibody is available. This potential issue does not seem to be preventing them from performing these quite interesting and valuable mapping experiments. Since they were able to rescue the ALX4 through over-expression, they should have the ability to put an affinity tag on the transgene, and this affinity tag could be used to map ALX4 location in the genome.

Minor issues:

line 124 - "suggest that... chromatin accessibility is essential is too strongly worded" They cannot justify using the word "essential".

Throughout the manuscript p-values for GO terms need to be adjusted for multiple testing - after adjustment, some term may no longer be significant.

**Reviewer 3:**

**Are you able to assess all statistics in the manuscript, including the appropriateness of statistical tests used?**

There are no statistics in the manuscript.

**Comments to author:**

In the current manuscript Jang et al describe the epigenetic landscape of zebrafish pigment cell differentiation. The authors generate DNA methylome, ATAC-seq and RNA-seq maps to study differentiation of NCCs to melanocytes and iridophores. The authors describe an overall loss of DNA methylation and an increase in open chromatin at major transitions which has previously been demonstrated during differentiation in numerous model organisms including zebrafish. Also, as demonstrated before, these changes were mostly localized to distal (intronic, or intergenic) regions thus reinforcing the common view that most regulatory dynamics during differentiation can be observed at enhancers rather than promoters. The authors have then utilized RNA-seq to develop gene regulatory networks, which resulted in the identification of the alx4a gene (previously associated with expression in pigmented cells), for which the KO has been generated and assessed for iridophore depletion. While I believe that this work can be useful to the zebrafish community working on pigment cell differentiation, it is fair to say that the manuscript has little novelty to offer.

Much more could have been done with RNA-seq data. For example, it is currently not clear how changes in ATAC-seq / WGBS relate to transcriptional changes (except for DEG numbers). Also, ChIP-seq for alx4a would perhaps make this manuscript more exciting as it would allow the authors to verify their network predictions.

Minor comments:

I would avoid phrases like: "immense mutagenesis", "herculean forward genetic experiments" and others.

The representation of WGBS data in fig1 - it is very difficult, if not impossible, to see CpG sites and CpG coverage.

**Authors Response**

**Point-by-point responses to the reviewers’ comments:**

*General Responses*

*We are extremely grateful for the reviewers' careful consideration and constructive comments to improve the quality of the work presented in this manuscript. Similar to the experiences of many others, especially throughout the pandemic, this particular journey has been a rough roller-coaster ride. But we are so encouraged by the reviewers' positive receipt of this work and look forward to addressing the comments raised below. Through the additional bioinformatic analysis and the clarification of language used within the manuscript, we believe that we have resolved all of the concerns that the reviewers raised, except the experiment of characterizing alx4a binding profile in iridophores. We pointed out that the lack of a high-quality the alx4a antibody prevented us from generating the alx4a ChIP-seq profile. Reviewer 2 provided an attractive alternative experiment idea of generating a transgenic fish with alx4a-transgene expressing an affinity tag to act as a surrogate for pull-down enrichments. This is an ingenious idea that completely slipped our mind and something that we are excited to look into! However, we believe that these additional nontrivial experiments might not fit the scope of this paper, which is to highlight the power of comparative epigenetics to generate mechanistic hypotheses and identify candidates that drive previously under-characterized cell type differentiation. Making an affinity-tagged TF transgenic animal, validating it and performing all the genomic experiments are exciting but will drastically delay the publication of our current results, which is not dependent on the alx4a genome-wide binding profile. Indeed, our comparative epigenetic work on iridophore differentiation in zebrafish has provided numerous exciting candidates for follow-up validation and mechanistical studies. We would like to propose, if reviewers are willing, to publish the improved manuscript without the inclusion of the alx4a binding profile, which will be provided in an independent follow-up study looking at binding profiles of multiple alx TFs and gbx2 in zebrafish iridophores to dissect the genetic and epigenetic dependencies that drive iridophore differentiation.*

*We have marked the major edits in the main text in our revised manuscript: the new text is marked with underline, while the removed text is marked with a strikethrough.*

*Below, we provide the point-by-point responses to each Reviewer's comments.*

POINT-BY-POINT RESPONSES

Reviewer #1: The manuscript by Jang et al. presents a substantial amount of meticulously collected and analyzed data about the epigenetic progression of pigment cell differentiation in zebrafish. Starting with 4 purified cell populations, they track changes in gene expression, methylation, and chromatin accessibility through the early stages of neural crest cell commitment and the formation of two pigment cell types, melanophores and iridiphores, that form from a common pigment precursor cell. They present the analyses clearly, and many of their findings confirm known aspects of NCC and pigment cell differentiation. Importantly, they also reach a number of significant and novel conclusions. First, they look specifically at the overlap of differentially methylated regions (DMRs) and differentially accessible regions (DARs), so-called DMARs. These are not only better correlated with nearby differentially expressed genes (DEGs) but also tend to correspond to evolutionarily conserved regions. Although they emphasize the finding in the context of iridiphores, it is likely generalizable to many cell differentiation processes. Second, they find that the promoters of DEGs do not show correlative changes in either methylation or accessibility, but instead the correlative DMARs are located in intergenic and intronic regions, suggesting that the gene expression changes driving differentiation are regulated through distal enhancers, not promoters. Finally, they use motif analysis of the DMARs to identify potential regulatory transcription factors, and implicate the alx gene family. They confirm the functional significance by generating mutants of all alx genes, showing that alx4a mutants specifically lack iridiphores. Furthermore, ectopic expression of alx4a, and the previously identified iridiphore gene gbx2, prevent melanocyte formation, presumably by driving development of the common pigment precursor to the iridiphore lineage.

The data and the analysis are thorough and high quality. The conclusions are well-supported by the data. If anything, the authors have undersold their work. I have only a few minor comments:

*(1.0) We are grateful to the reviewer for the positive comments and highlighting the power behind comparative epigenetics that can be generalizable to many other cell differentiation processes!*

1. In the title and throughout, I would argue that melanophore is the correct term, not melanocyte.

*(1.1) In the Johnson lab, we often used melanocytes and melanophores interchangeably to describe black/brown pigment cells. However, the reviewer's argument is correct in that melanophores are more broadly applied to describe black/brown chromatophores in mammal, birds and fish. The term "melanocyte" describes the melanin-producing cells in the skin but is often applied to mammals and birds. We have updated the manuscript to replace the term "melanocyte" with "melanophore". We thank the reviewer for providing a more accurate terminology for the melanin-producing pigment cells in zebrafish.*

2. On the title page, the superscript indicates that Ting Wang is deceased (!), which I am certain must be a mistake, since he is the corresponding author.

*(1.2) We are horrified to have made this mistake! Indeed, the corresponding author (constructing this rebuttal) can attest that he is still alive and kicking. We thank the reviewer for catching the egregious mistake! We continue to pay our deepest respect to Professor Stephen Johnson who passed away in 2017.*

3. There are several typos in the reference list. Most are minor and would be caught in final editing, but #14 did not have enough information to make it possible to find the paper.

*(1.3) We are extremely grateful of the reviewer's keen eye to details. We have reviewed the reference list and we hope we have caught all of the typos.*

4. In Supp Fig 3a, the colors in the overlapping peaks are very hard to see - perhaps patterns would work better, or just showing the outline of the curves instead of filling them in?

*(1.4) We thank and agree with the reviewer that the overlapping peaks are hard to distinguish from each other. We made the appropriate changes as advised:*

Reviewer #2: Authors of Jang et al. state that the epigenetic landscape contributing to pigment cell differentiation is poorly understood. To investigate this, they charted the global changes in the epigenetic landscapes, DNA methylation and chromatin accessibility, during neural crest differentiation into melanocytes and iridophores, with the goal to identify epigenetic determinants. They use crestin:GFP as a marker to separate NCC stages and then did WGBS-Seq, RNA-Seq, and ATAC-Seq. This leads them to ALX4, and they then perform experimental studies to test ALX4 function in zebrafish.

This is a well written manuscript with significant results. The experiments presented in figures 3 and 4 are quite interesting and appear conclusive. While I find the overall story compelling, I have major issues with the initial epigenetics studies which led to their investigation of ALX4. These issues, mostly concerning figure 2, cause the manuscript to be very disjointed and confusing.

*(2.0) We thank the reviewer for the cautious enthusiasm for our work, constructive criticism and suggestions, and the opportunity to expand on the analysis of the epigenetic dynamics and how it correlates with gene expression. We hope that the additional epigenetic and GO-enrichment analysis and improving the clarity of the writing provide a better transition from global epigenetic analysis to the motif discovery and experimental validation of the necessity and sufficiency of alx4a for iridophore differentiation.*

Issues:

WGBS-Seq data as presented in figures is near impossible to interpret - Fig 1b specifically. Please separate the panel with CpG sites so that methylation levels can be seen clearly. Statement "associated with low promoter methylation and high chromatin accessibility" is not supported. This statement requires a statistical test comparing these regions, and I do not see this test in the manuscript.

*(2.1) We apologize for the issues with the WGBS-seq figures! The issue was created by the figure conversion into pdf which led to the black outlines to thicken. We have removed those black lines surrounding the CpG locations, which we hope improves the interpretability of the WGBS-seq figures (Response Fig. 2). We also specify in the figure legends that the each grey bar indicates the location of a CpG and that the height of the blue foreground represents DNA methylation level.*

*Furthermore, we apologize for the confusing language in "associated with low promoter methylation and high chromatin accessibility." The intention behind this phrase was to highlight that the promoters of the marker genes show low methylation and chromatin accessibility in Fig. 1b. We did not intend to globally attribute this phenomenon to all promoters or claim statistical significance of this phenomenon. To reflect our intended meaning, we highlighted the promoter regions with a red box in the figure and changed the language to: " Indeed, these marker genes are highly expressed and the promoters of these marker genes (red box, Fig. 1b) show low methylation and high chromatin accessibility in specific cell types."*

For DMR calling, biological replicates were combined to improve coverage of CpGs, but this approach may lead to increased false positive DMRs. Authors should evaluate identified DMRs measuring methylation for individual replicates to determine whether DMRs might be caused either by low read coverage or high methylation variability. If necessary, further filtering of DMRs should be applied to eliminate false positives.

*(2.2) We thank the reviewer for bringing up this extremely important and underappreciated aspect of DMR calling with various bioinformatic tools. We completely agree with the reviewer that there is a trade-off between combining biological reps (BRs) and analyzing them separately, where combining BRs can lead to low specificity. We had the same concerns thus compared biological significance of various comparisons and concluded that combining biological replicates and using a stringent p-value cutoff of 0.01 seem to provide best balance of sensitivity and specificity. Below, we go into more detail on the comparison of DMRs provided in the manuscript and the DMRs called by DSS by using two BRs.*

*First, we characterized the number of DMRs detected between two biological replicates of the same cell type. The number of DMRs ranged from 241-420 DMRs at a p-value cutoff of 0.01, which provides the rough estimate of the false-positive detection rate of the DSS algorithm. Next, we compared the number of DMRs detected when biological replicates were processed separately or combined. As expected, we observed higher number of DMRs when separate BRs were used with a p-value cutoff of 0.05 than with a p-value cutoff of 0.01. When we called DMRs using combined BRs with a p-value cutoff of 0.01, we observed a similar number of DMRs with the number detected using separate BRs with the same p-value cutoff. Over 95% of the DMRs are shared between separate BR DMRs (p-value <0.01) and combined BR DMRs (p-value <0.01), indicating that the DSS detection of DMRs is consistent whether you combine or keep the BRs separate (Response Fig. 3b). We observed a lower proportion of DMRs being shared in early NCC vs. late NCC comparison, but the number of DMRs falls within the range of false-positive DMRs. Therefore, the lower overlap in DMRs (between the two methods with separate and combined BR) in early NCC vs. late NCC could be due to the relatively higher number of false-positive DMRs in the comparison.*

*Furthermore, we looked more closely at the DMRs that were detected in the separate BR DMRs (p-value <0.05), but missing from combined BR DMRs (p-value <0.01). As the reviewer suggested, we looked at the coverage distribution of the DMRs that were unique to separate BR DMRs or shared between the two categories. The distribution of coverage of unique and shared DMRs was remarkably similar (Response Fig. 4a). Instead, we noticed that the unique DMRs have lower CpG counts per DMR (Response Fig. 4b). The difference in methylation in these sparse CpG DMRs is considered to be lower in statistical significance and filtered out when filtering for p-value <0.01. Whether sparse CpG DMRs have significant biological consequence is unclear. Therefore, between two DMR sets called with the combined and separate biological replicates, we observed that the >95% of DMRs are shared under the same p-value cutoff. Therefore, we decided to use a more stringent p-value cutoff to look at DMRs with higher CpG counts, which we believe might have higher functional/gene regulatory potential in cell differentiation. In conclusion, combining the biological replicates did not significantly increase the number of false positive DMRs when compared to DMR detection using biological replicates separately.*

At line 113 - what is meant by "these shared DMRS could predict the DNA methylation landscapre of the intermediate pigment progenitor cell"?... do they mean that DMRs have this potential, or can DMRs were actually do this, because I find nowhere in the manuscript that authors attempted to make unsupervised predictions based on methylation status?

*(2.3) We agree with the reviewer that this statement is a bit vague and inconclusive with the data provided in the manuscript. The intention behind the statement was to suggest that these shared hypoDMRs could be regulating shared biological processes, such as cell migration or general pigmentation pathways, that occur in a bipotent pigment progenitor cell (also known as melanoiridoblast). Indeed, when we performed gene ontology enrichment of the closest differentially expressed gene within 50kb of the shared hypoDMRs in melanophores and iridophores, we report gene ontology terms such as "pigmentation", "ameboidal-type cell migration", and “embryonic development” as top enrichment hits (Response Fig 5). Although these results support our claim that these shared hypoDMRs could be of melanoiridoblast origin, we agree that this is still not a strong enough support that shared hypoDMRS are present in melanoiridoblast. The most conclusive support for this claim would be to perform single cell DNA methylation profiling of neural crest differentiation. Therefore, we decided to remove the melanoiridoblast statement and instead focus on the claim that these hypoDMRs might regulate neural crest migration and pigmentation gene expression. To reflect this change, we replaced the original sentence with "These shared hypoDMRs enrich near differentially expressed genes related to neural crest migration ("ameboidal-type cell migration"32) and pigmentation (Fig. S1j), suggesting that DNA methylation could regulate neural crest differentiation into pigment cells."*

Authors indicate they focused on "dynamic" DMARs, but how do they define "dynamic" and in what manner did they focus on these regions? In figure S4 they begin analysis of what might be dynamics regions, comparing methylation levels at different stages in open or closed regions, but there is no downstream analysis. Analysis in figure S5 is not sufficient. What type of biologically relevant information can be gleaned from separate types/clusters of "dynamic" DMAR regions?

*(2.4) We again apologize for the lack of a clear definition for the terminologies used in the manuscript. Our definition of "dynamic" was regions undergoing both methylation and chromatin accessibility change across differentiation. We believe that the term "dynamic" does not add additional value or clarity but could confuse for the readers; thus we decided to remove that term. We thank the reviewer for clearing up the ambiguous language in our manuscript!*

*To provide more biologically relevant information of these epigenetically dynamic regions (regions with DNA methylation and/or chromatin accessibility changes), we performed Gene Ontology enrichment of DEGs near these regions. This analysis provided interesting insights into how various biological processes might be controlled by specific epigenetic marks.*

*One striking observation was that GO terms that enriched for hypoDMRs and solo closingDARs had high concordance with GO terms enriched in down-regulated genes across neural crest differentiation into pigment cells (Response Fig. 6). One interpretation of this data is that DNA methylation plays a role in defining the early phases of NCC differentiation, further demarcated by loss of chromatin accessibility after pigment cell differentiation. In contrast, melanophore-specific and iridophore-specific terms important for cell type-specific physiology were only enriched in shared openingDARs and hypo-openingDMARs (Response Fig. 7). In conclusion, we believe that this analysis strengthens the biological connection between DEGs and the potential cis-regulatory roles of epigenetically dynamic regions identified through comparative epigenetic analysis.*

The general and somewhat broad characterization of differentially accessible and differentially methylated regions in figure 2 seems disjointed and unfocused. I would have much preferred more focused analysis investigating the overlap of DARs and DMRs, including in depth characterization of regions that meet the general expectation - regions becoming open also lose DNA methylation and closed regions become methylated - but authors fail to investigate regions to do not meet this expectation. For example, authors indicate that 4000/6000 iridophore-specific solo hypoDMRs occur at closed regions, but they do not investigate these regions further. If the genome is generally becoming more demethylated as late NCCs differentiate, then why are most regions less accessible? I would have expected the opposite. They make note of this issue in lines 138-140, but they do not investigate it more deeply.

*(2.5) We thank the reviewer for the constructive advice! To compare the levels of ATAC-seq signal and DNA methylation levels across DMRs, DARs, and DMARs, we generated a line graph representing DNA methylation level and heatmap representing ATAC signal (Response Fig. 8a). These figures shows apparently that the majority of the solo hypoDMRs have relatively low levels of ATAC-seq signal. In addition, solo hypoDMRs have fewer CpG counts, and are smaller regions when compared to DMARs (Response Fig. 8b). These results suggest that the hypoDMRs are not accessible thus might not retain cis-regulatory function. Furthermore, we note that the average level of DNA methylation decreases when looking at solo openingDAR thus could have more impactful cis-regulatory roles relative to hypoDMRs. Indeed, DEGs near DMRs show a weak correlation with gene expression change, while DEGs near openingDARs and hypo-opening DMARs show an increase in gene expression and vice versa for closingDARs and hypo-closing DMARs (Response Fig. 8c). We believe these results provide correlative support that the epigenetically dynamic regions do impact nearby gene expression levels. We incorporated this new discovery into the manuscript and are grateful to the reviewer for this great idea!*

At line 157, authors performed GO analysis on DMARs and state that "For both melanocyte and iridophore DMARs, numerous essential neural crest... were enriched" It seems in figure 2E that the most enriched pathways are for general developmental pathways, and not NCC-specific pathways. Authors should adjust their wording to correct this issue. They do identify pigment cell and neural crest cell related GO terms when they performed GO analysis based on gene expression levels, but the question remains - how do accessibility changes and DNA methylation changes relate the expression changes?

*(2.6) We agree with the reviewer that the previous GO analysis provided too general aspects of developmental pathways. In the revised manuscript, we performed GO enrichment by taking the closest DEGs within 50kb of DMR/DAR/DMARs. We also performed multiple testing correction and filtered GO terms for q-value <0.05 as requested in the reviewer's minor issue #2. Please refer to Response 2.5, which addressed how various DMRs, DARs, and DMARs affect nearby DEG gene expression.*

These issues with initial characterization or epigenetics data make the transition to figure 3 quite difficult and abrupt. The remaining portion of the paper rests completely on their identification of an enriched motif. I would have been nice if the authors measured epigenetic changes (DNA methylation and accessibility) relative ALX4 motifs across the genome. This would help the reader understand how general and dynamic ALX4 binding might be during iridophore differentiation. Once they've identified ALX4, the paper moves in a completely different and interesting direction, almost making the first half of the paper seem irrelevant. More in-depth analysis of epigenetic changes relative to ALX4 would help the reader understand how epigenetic factors may influence ALX4, and also help to connect the first half of the paper with the second half.

*(2.7) We sincerely hope that the aforementioned proposed amendments in supplementing the epigenetic dynamics with gene expression have provided some clarity and a smoother transition into the motif enrichment analysis. We agree with the reviewer that the sudden jump from motif detection to function validation through genetic experiments could cause some whiplash to the reader. We are grateful for the reviewer's perceptive recommendation to tie in ALX4 motif presence and relate it to the epigenetic dynamics that determining iridophore cell fate.*

*Before diving into the analysis, we want to note that, through careful code review, we recognized that the ALX and GBX2 motifs used for the FIMO DM/AR detection were not identical with the motif used in CENTIPEDE for the footprint analysis. We have remedied this issue by rerunning the FIMO motif discovery using the correct ALX and GBX2 motifs (no changes were necessary for other TFs). This remedy led to slight changes in the quantification of DM/ARs with ALX or GBX2 motifs within 50kb of the guanine-related and GO-enriched DEGs. We have corrected in the manuscript to update to the correct number:*

*"Numerous iridophore-specific DEGs (82.4 70.6%) responsible for guanine generation and transport have at least one DMAR with alx motif (Fig. 3d, Fig. S8)."*

"74.6 76% of these DEGs have at least one DM/AR with an alx motif (Fig. S8), suggesting the putative regulatory potential of alx TFs for iridophore’s reflective characteristic."

*We respectfully apologize for this mistake!*

*To address the reviewer's recommendation, first, we investigated the frequency and distribution of iridophore-related DM/ARs with ALX, SOX10, GBX2 and TFEC motifs (transcription factors deemed to be essential for iridophore enrichment). We report that the ALX-containing DM/ARs were most abundant (Response Fig. 9a). In addition, ALX-containing DM/ARs were over-represented by hypo-openingDMARs (~3 fold greater than expected) and solo openingDARs (~2.2 fold greater than expected) while under-represented by solo closingDARs (~5.7 fold less than expected). In comparison, GBX2-containing, TFEC-containing, and SOX10-containing hypo-opening DMARS were only ~1.7, ~1.5, and ~1.9 fold greater than expected respectively. Next, we explored the DNA methylation and chromatin accessibility dynamics associated with the iridophore-associated epigenetically dynamic regions with ALX, SOX10, GBX2, and TFEC motifs. This analysis led to the discovery that a fraction of solo openingDARs and hypo-openingDMARs that contain SOX10 (17.2% & 29.5%), TFEC (19.6% & 41.5%), and GBX2 (17.6% & 17.9%) motifs were shared in the melanophore (Response Fig. 9b). In stark contrast, less than 6% and 14% of the solo openingDARs and hypo-openingDMARs, respectively, containing the ALX4 motifs were present in melanophores. In conclusion, the discovery that ALX-containing DM/ARs are most frequent and enrich for activating epigenetic dynamics (openingDAR or openingDMAR) and that most ALX-containing DMARs are specific to iridophores further corroborates the potential importance that the alx transcription factors might have to iridophore cell fate. We highlight these findings by restructuring Figure 3 to focus solely on motif discovery and the epigenetic changes associated with DM/ARs containing the enriched motifs. We reformatted Figure 4 to include all the functional validations performed in this manuscript. We hope this will help create a better transition from the epigenetic dynamics to motif enrichment to functional analysis in this manuscript.*

More details are needed to understand how authors generated "putative transcription factor network that drive iridophore cell fate" notes in line 184. Generally, this network seems somewhat interesting, but has little impact on the results of the manuscript. I suggest putting less emphasis on this result. Authors highlight this network in the abstract, and it sets the expectation that a bioinformatically established and experimentally validated network was constructed. These things were not done, and so I recommend eliminating this claim.

*(2.8) We apologize for the oversight in detailing how the putative transcription factor network was generated! We added another section in the methods describing how we generated the network: "Using motif enrichment results from iridophore-specific DMRs, DARs, and DMARs, we curated a list of previously-known transcription factors related to iridophore development (sox1010, foxd39,22, tfec23,24, and gbx225). In a murine model, Ets1 has been shown to be essential for Sox10 expression for proper melanocyte development81. Since ets1 was found to be enriched in iridophore DMARs, we decided to incorporate ets-1 and alx TF candidates into the putative transcription factor network. Using transcriptomic data, we ordered the TFs based on the presence and abundance of TF expression across NCC differentiation into iridophores (Response Fig. 10). Then we queried all DM/ARs within 50kb of TF promoters for the presence of various TF motifs using FIMO and created putative TF-TF connections to create the network."*

*Furthermore, we softened the language to remove the strong claim on the aspects of the transcription factor network in the abstract. "Motif enrichment in the epigenetically dynamic regions revealed putative transcription factors that might be responsible for driving pigment cell identity. Through this effort, in the relatively uncharacterized iridophores, we present a network of transcriptions factors that are predicted to bind to regulatory elements directly upstream of genes linked to the guanine synthesis cycle and validate alx4a as a necessary and sufficient transcription factor for iridophore differentiation and present evidence on alx4a's potential cis-regulatory role in guanine synthesis pathway."*

Indeed, the re-expression of ALX4 rescues iridophore differentiation in mutant fish, but re-expression also seems to rescue the uncharacterized melanocyte phenotype - why do the broken lateral stripes in mutants become more normal continuous stripes in the ALX4 over-expression fish?

*(2.9) Great attention to detail! Although we did not really expand on the biology behind proper stripe formation in zebrafish, iridophore has been shown to play a crucial role in stripe formation in the skin of zebrafish body. Multiple groups have already shown that mutants deficient in iridophores have showed a strong reduction of melanophore numbers and defects in stripe formation1,2,3. More importantly, transplantation of iridophore to iridophore-deficient mutants restored stripe formation in the skin of zebrafish body1: They. transplanted nac;pfe mutant cells, where iridophore is the only chromatophore, into rse or shd mutants zebrafish that are deficient in iridophore and have abnormal stripe formation. The generated chimeric animals displayed a large region of restored stripe formation similar to the rescue that the reviewer astutely pointed out in the chimeric alx4a-rescue fish. Frohnofer et al. have also shown that body stripes of the transplanted locations were restored in the reciprocal experiment. A similar phenotype of stripe restoration was found from the research of Krauss et al.2.. They showed that tra mutants display defects in iridophore maintenance and stripe formation. To test if the interaction between iridophores and melanophores is important for pigment stripe pattern formation, they introduced wild-type iridophores into mutants by transplanting nac;pfer double mutants cells to provide wild type iridophores to tra mutants. The transplantation indeed restored the normal stripe formation, which was further confirmed in the reciprocal experiment. Another indirect evidence is from the research of Patterson and Parichy3, in which, bnc2 mutants were found to exhibit far fewer melanophores, xanthophores and iridophores. Rescue of melanophores and xanthophores by kitlga and csf1 in bnc2 mutants respectively was insufficient to recover stripes formation in the bnc2 mutant. Overall, we are encouraged to see the rescue of the stripe formation, which is consistent with work from other labs. We briefly touch upon this point in the manuscript: “Two unique alx4a mutant alleles displayed clonal iridophore rescue and reestablishment of proper lateral stripe formation51-53, suggesting that re-expression of alx4a during late NCC stage was sufficient to reestablish proper iridophore differentiation (Fig. 4f, Fig. S11c).”*

*References:*

*1. Frohnhöfer, Hans Georg, et al. “Iridophores and Their Interactions with Other Chromatophores Are Required for Stripe Formation in Zebrafish.” Development, no. 14, The Company of Biologists, July 2013, pp. 2997–3007. Crossref, doi:10.1242/dev.096719.*

*2. Krauss, Jana, et al. “Transparent, a Gene Affecting Stripe Formation in Zebrafish, Encodes the Mitochondrial Protein Mpv17 That Is Required for Iridophore Survival.” Biology Open, no. 7, The Company of Biologists, June 2013, pp. 703–10. Crossref, doi:10.1242/bio.20135132.*

*3. Patterson, Larissa B., and David M. Parichy. “Interactions with Iridophores and the Tissue Environment Required for Patterning Melanophores and Xanthophores during Zebrafish Adult Pigment Stripe Formation.” PLoS Genetics, edited by Gregory S. Barsh, no. 5, Public Library of Science (PLoS), May 2013, p. e1003561. Crossref, doi:10.1371/journal.pgen.1003561.*

Authors state that they would like to map ALX4 localization in the genome, and they cannot do this until an antibody is available. This potential issue does not seem to be preventing them from performing these quite interesting and valuable mapping experiments. Since they were able to rescue the ALX4 through over-expression, they should have the ability to put an affinity tag on the transgene, and this affinity tag could be used to map ALX4 location in the genome.

*(2.10) As mentioned in the General Responses, we are extremely grateful for this ingenious idea of overcoming the lack of specific alx4a antibodies for ChIP-seq experiments. We completely agree with both Reviewer 2 and Reviewer 3 that alx4a ChIP-seq would add incredible value to the understanding of the mechanism by which alx4a regulates iridophore differentiation. However, our goal with this paper was to provide high-quality epigenetic data across NCC development into melanophores and iridophores, which were sorely missing in the field. We also wanted to highlight the utility of comparative epigenetics as a method to identify important gene regulatory networks that drive cell fate, which led to the discovery of alx factors. Although the alx4a has been shown to be an iridophore marker gene, no functional validation or the wide breadth of information on potential binding sites and potential cis-regulatory insights have been published in a peer-reviewed journal. Furthermore, we discovered that the loss of other iridophore marker genes (alx1, alx3, and alx4b) are not essential for iridophore differentiation. We hope that the nontrivial amount of work presented in this paper will prevent others from performing time-consuming genetic ablation experiments. Lastly, our hope is to follow-up this manuscript with molecular techniques to understand the grammar and the epigenetic influences of alx4a and gbx2 in iridophores. We hope to generate a tagged alx4a-transgenic animal, validate that the tagged TF has the wild-type function, and map its genome-wide localization in a separate, independent study.*

Minor issues:

line 124 - "suggest that... chromatin accessibility is essential is too strongly worded" They cannot justify using the word "essential".

*(2.11) We agree with the reviewer and have removed this phrase to soften the claims in the paper.*

Throughout the manuscript p-values for GO terms need to be adjusted for multiple testing - after adjustment, some term may no longer be significant.

*(2.12) We thank the reviewer for improving the rigor of the analysis provided in this manuscript. We have regenerated all the GO enrichment plots to present terms that pass the q-value cutoff of 0.05.*

Reviewer #3: In the current manuscript Jang et al describe the epigenetic landscape of zebrafish pigment cell differentiation. The authors generate DNA methylome, ATAC-seq and RNA-seq maps to study differentiation of NCCs to melanocytes and iridophores. The authors describe an overall loss of DNA methylation and an increase in open chromatin at major transitions which has previously been demonstrated during differentiation in numerous model organisms including zebrafish. Also, as demonstrated before, these changes were mostly localized to distal (intronic, or intergenic) regions thus reinforcing the common view that most regulatory dynamics during differentiation can be observed at enhancers rather than promoters. The authors have then utilized RNA-seq to develop gene regulatory networks, which resulted in the identification of the alx4a gene (previously associated with expression in pigmented cells), for which the KO has been generated and assessed for iridophore depletion.

While I believe that this work can be useful to the zebrafish community working on pigment cell differentiation, it is fair to say that the manuscript has little novelty to offer.

*(3.0) We thank the reviewer for the positive comments about this manuscript! Considering that very minimal epigenetic data is available for pigment cells, especially iridophores, we hope that the epigenetic data provided here would help those in the zebrafish pigment community. The inspiration behind this study was driven by Professor Stephen Johnson's desire to better characterize the epigenetic dynamics that drive pigment cell fate and we are proud to have been part of his zebrafish legacy. By comparing the epigenetic data generated through this effort, we were able to identify putative transcription factors that might be relevant to melanocyte and iridophore cell fate. Although we were able to replicate many past discoveries using this orthogonal method, we also provide novel putative candidates that have not been published before. However, the reviewer is correct that alx4a has been known to be highly expressed in iridophores specifically (previous work from Steve Johnson lab). Still, we argue that no published work is available that validated the necessity of this TF. Furthermore, other alx TFs are preferentially expressed in iridophores. We generated numerous KO lines for alx TFs and reported that only alx4a is essential for iridophore development on zebrafish body, but not the eye. We believe that the negative result of alx1, alx3, and alx4b raises an interesting biological question about their role in iridophore development, but also provide a resource to the zebrafish community to avoid duplicate and time-consuming work. Furthermore, we performed rigorous subsequent experiments to really nail down that alx4a loss is responsible for iridophore ablation by reintroducing alx4a expression in KO fish and showing iridophore rescue. Lastly, we engineered stable lines that contain a transgene that expresses alx4a or gbx2 behind a mitfa promoter to show that ectopic expression of these two TFs in early NCC can push pigment cell fate to favor the production of iridophores over melanophores. Overall, we believe that this work provides (hopefully) valuable epigenetic resources and rigorous scientific validation of alx TFs’ role on iridophore development.*

Much more could have been done with RNA-seq data. For example, it is currently not clear how changes in ATAC-seq / WGBS relate to transcriptional changes (except for DEG numbers). Also, ChIP-seq for alx4a would perhaps make this manuscript more exciting as it would allow the authors to verify their network predictions.

*(3.1) Please refer to Response 2.4, 2.5, and 2.6, which provide deeper insights on the potential biological functions of these epigenetically dynamic regions and how they potentially impact gene expression levels throughout pigment cell differentiation. For response to alx4a ChIP-seq recommendation, please refer to the General Responses and Response 2.10.*

Minor comments:

I would avoid phrases like: "immense mutagenesis", "herculean forward genetic experiments" and others.

*(3.2) We apologize for using over-sensationalized words in the manuscript. We have attempted to soften the tone in the manuscript and removed the term "immense" and "herculean".*

The representation of WGBS data in fig1 - it is very difficult, if not impossible, to see CpG sites and CpG coverage.

*(3.3) We are shocked and apologetic to have missed this issue with the figure! We have changed the figures and amended the figure legend to easier interpretability of the WGBS browser view. Please refer to Response 2.1 for more information.*

**Second round of review**

**Reviewer 1**

**Reviewer 2**

While there are some aspects of the manuscript that are improved, I do not find the manuscript has properly addressed my concerns. I have major issues with the bioinformatics methods used to assess differentially methylation regions and differentially accessible regions. Their non-standard approach leads me to question whether any of their conclusions relative to the genomics analyses can be trusted. The manuscript presents largely qualitative measures using percentages, amounts, and aggregate measurements, with statistical tests limited almost exclusively to GO analysis. Data contributing to figures 1 and 2 need to be completely re-analysed using more rigorous approaches, including statistical tests based on biological replicates and model-based testing to defining particular regions of interest - common methods include DiffBind, DeSeq2. Authors wold also need to perform multiple testing correction once these approaches are utilised. The authors could have easily accommodated these concern in revision, but they regularly chose to re-word their conclusions rather than being more rigorous with their analyses, which is even more concerning.

As I indicated in my prior review, I do find figures 3 and 4 to be quite interesting, and the functional data is quite compelling. In this context, I find figures 1 and 2 to be almost irrelevant. Here again however, the authors chose not to perform a highly feasible experiment... measuring ALX4 genomic localisation.

Unfortunately I can only recommend that this manuscript undergo a major revision prior to publication in Genome Biology.

**Reviewer 3**

The authors have submitted an improved version of the previous manuscript. The figures are easier to understand and a substantial amount of analyses have been added to the paper. Nevertheless, the paper still offers little novelty. For example, there is nothing particularly interesting regarding the epigenetic dynamics of pigment cell differentiation in zebrafish. Also, the authors have not made an attempt to identify genomic binding sites of alx4a or to try and experimentally validate the proposed network. Overall, I think that such a manuscript might appeal to a small number of researchers working on pigment cell differentiation in zebrafish who might find the associated datasets useful in future studies. Also, I find it odd that the data have not yet been submitted to GEO and that the code has not been made available in supplementary data or on a github page. The authors state: "Code used for analysis will be available upon request".

**Authors Response**

**Point-by-point responses to the reviewers’ comments:**

Point-by-point Responses

*We thank the reviewers for providing a second round of comments and critiques. As both reviewers mentioned, we believe we significantly revised and improved the quality of the manuscript from the previous version. Reviewer 3 states that “The authors have submitted an improved version of the previous manuscript. The figures are easier to understand and a substantial amount of analyses have been added to the paper” to highlight the tremendous amount of additional analysis that was performed to answer the reviewers’ previous concerns. Here, we address the concerns raised in the second round of review.*

*We have marked new additional edits in the main text of the re-revised manuscript: the new text is marked with underline, while the removed text is maker with a strikethrough.*

Reviewer 2: While there are some aspects of the manuscript that are improved, I do not find the manuscript has properly addressed my concerns. I have major issues with the bioinformatics methods used to assess differentially methylation regions and differentially accessible regions. Their non-standard approach leads me to question whether any of their conclusions relative to the genomics analyses can be trusted. The manuscript presents largely qualitative measures using percentages, amounts, and aggregate measurements, with statistical tests limited almost exclusively to GO analysis. Data contributing to figures 1 and 2 need to be completely re-analysed using more rigorous approaches, including statistical tests based on biological replicates and model-based testing to defining particular regions of interest - common methods include DiffBind, DeSeq2. Authors wold also need to perform multiple testing correction once these approaches are utilised. The authors could have easily accommodated these concern in revision, but they regularly chose to re-word their conclusions rather than being more rigorous with their analyses, which is even more concerning.

*(2.1) Again, we appreciate Reviewer 2’s critique for statistically rigorous bioinformatic methods. Reviewer 2 claims that our method is “non-standard approach” and that we should use “statistical tests based on biological replicates and model-based testing to defining particular regions of interest – common methods include DiffBind, DeSeq2”.*

*First, we are unsure how to interpret what Reviewer 2 means by "non-standard approach". Our lab has been fortunate enough to have participated and led multiple large NIH funded genomic consortia (Roadmap Epigenomics, ENCODE, 4D Nucleome, TaRGET, Human Pangenome Reference Consortium), where we helped establish the bioinformatics standard together with numerous leaders in the field. Our lab prides ourselves in generating high-quality data and performing statistically rigorous bioinformatic analysis. This effort often results in testable hypothesis that provides meaningful biological insights, which we validate with functional genomic studies. This manuscript is no exception to the high standard we hold for ourselves.*

*As detailed in Methods section of the manuscript, we generated an array of WGBS, mRNA-seq, and ATAC-seq libraries of each cell type in two biological replicates (two independent experiments performed on different days). To call differential methylation, accessibility and gene expression, we abided to the standard workflow of the “common methods” (as defined by Reviewer 2) to call statistically significant candidate with few exceptions detailed below.*

*For differential methylation region (DMR) detection, we used the DSS tool, which is one of the highly cited differential methylation statistical tools and allows rigorous statistical comparison of each CpG methylation across samples. The DSS group also published the utility and accuracy of DSS using smoothed methylation values without replicates (Wu et al. 2015). We followed DSS standard workflow, but made one change to further increase sensitivity and confidence of the DMR detection: “the DSS tool with standard parameters plus “smoothing=TRUE, delta=0.30 (at least 30% methylation difference), and p.threshold=0.01”. We stringently filtered DMRs for p-value < 0.01 (using Wald test) instead of the standard p-value <0.05. DSS standard workflow performs filtering on p-values instead of FDR, which is what we utilized for filtering DMRs based on statistical significance. In our Supplemental Figure 1, we established the replicability of global methylation landscape across the cell type biological replicates to justify combining the biological replicates into one for downstream analysis. We reasoned that having higher coverage of CpG can potentially increase accuracy of each CpG methylation calls. Reviewer 2 requested to look at DMRs called when biological replicates were compared independently instead of combined. As we show previously in Response Figure 3 of the 1st rebuttal response, we illustrated that ~95% of the DMR called are shared between analysis done with replicates separated or combined. Therefore, we reasoned that the DMR calls are not significantly changed whether the biological replicates are separated or combined and that the DMR calls are statistically sound.*

*For differentially expressed gene (DEG) analysis, as detailed in Methods section, we have already used the DESeq2 (as recommended by Reviewer 2) to call statistically significant genes with these metrics: “More specifically in DESeq2, we identified significantly differentially expressed genes by filtering for only genes with counts >1, fold change >2 and p-value < 0.01”. Again, to improve sensitivity of DEG detection, we filtered DEGs using adjusted p-value < 0.01 instead of the standard < 0.1 metric. The p-values provided by DESeq2 are derived by Wald test and corrected for multiple hypothesis testing with Bonferroni adjustment. However, we recognize that we used the incorrect term "p-value" when the correct term should be "adjusted p-value" in the Methods section. We apologize and have correct this mistake!*

*For differential chromatin accessibility analysis, we followed ENCODE standards of performing MACS2 (calling peaks), irreproducible discovery rate framework (identify consensus peaks), and DiffBind (statistical tool for calling differential peak). Again, as detailed in our Methods section, we analyzed the ATAC-seq data with DiffBind and identified DARs using an extremely stringent FDR cutoff (multiple hypothesis corrected) value of 0.001 instead of the standard 0.05 or 0.01 cutoff.*

*The data presented in Figure 1 and 2 are the quantitative (and qualitative) results of statistically rigorous detection of differential methylation, accessibility and gene expression. This was acknowledged by Reviewer 1: “The data and the analysis are thorough and high quality. The conclusions are well-supported by the data.”*

*With this response, we hope that we have adequately addressed Reviewer 2’s major concern on the rigor of our statistical bioinformatic analysis. To reiterate, the recommended bioinformatic analysis that Reviewer 2 suggested has already been performed with more stringent statistical cutoff for this manuscript (as detailed in Methods section). To help prevent any future miscommunication, we have provided the bioinformatic tool and the statistical cutoff utilized in the Results section (lines 96-97, 107-109, and 109 in the manuscript) and also in the Figure 1 legend. Furthermore, we have made available the code/scripts used for this analysis on GitHub (https://github.com/yjchen1201/zebrafish_pigment_cell_dev).*

*We hope that we have adequately addressed Reviewer 2's concerns about the bioinformatic methods and humbly ask for the re-evaluation of the revised manuscript with this clarification in mind.*

As I indicated in my prior review, I do find figures 3 and 4 to be quite interesting, and the functional data is quite compelling. In this context, I find figures 1 and 2 to be almost irrelevant. Here again however, the authors chose not to perform a highly feasible experiment... measuring ALX4 genomic localisation.

*(2.2) We thank the Reviewer 2 for the kind remarks on the functional validation aspect of this paper. The statistically rigorous analysis of the epigenetic and transcriptomic landscapes provided confident candidates that might be responsible for melanophore and iridophore differentiation. We absolutely agree that alx4a binding profile will be valuable. We plan to conduct an independent study to compare binding profiles of all alx4 factors and gbx2. However, this proposed new study will be much more focused on pigment cell differentiation, and much less on illustrating a new and general paradigm, which is what our current manuscript is about (and why we submitted to Genome Biology rather than a zebrafish-specific journal). Importantly, the new experiments will take a significant amount of time to conduct properly – make transgenic fish, validate, and repeat all genomic experiments. We certainly do not want to rush these experiments and lose the scientific rigor that we highly value. Furthermore, we hold steadfast in our belief that alx4 binding profile should not make or break our current manuscript. Whether alx4a binding profile is provided or not, the main point of the paper (the power of comparative epigenetics in zebrafish) stands strong and has been rigorously validated through laborious genetic experiments in this manuscript. Again, we would like to reemphasize that the main purpose of Figures 3 and 4 was to highlight and validate the power of comparative epigenetics for identifying high-confidence candidates. Figures 1 and 2 are essential to set the stage on how we identified these confident candidates for follow-up validation. We believe all these figures are necessary to create a coherent story that the readers of Genome Biology will appreciate. In conclusion, we graciously request for the reconsideration of the adamant necessity and requirement of the alx4 binding profile for this paper, especially in the context of Genome Biology's benevolent COVID policy.*

Unfortunately I can only recommend that this manuscript undergo a major revision prior to publication in Genome Biology.

*(2.3) We have already performed the bioinformatic analysis to higher rigor and statistical stringency than what Reviewer 2 recommended. We sincerely hope that this was an accidental oversight by Reviewer 2.*

Reviewer 3: The authors have submitted an improved version of the previous manuscript. The figures are easier to understand and a substantial amount of analyses have been added to the paper. Nevertheless, the paper still offers little novelty. For example, there is nothing particularly interesting regarding the epigenetic dynamics of pigment cell differentiation in zebrafish. Also, the authors have not made an attempt to identify genomic binding sites of alx4a or to try and experimentally validate the proposed network. Overall, I think that such a manuscript might appeal to a small number of researchers working on pigment cell differentiation in zebrafish who might find the associated datasets useful in future studies.

*(3.1) We are appreciative of Reviewer 3’s recognition of the amount of additional work that went into the revision. Reviewer 3 critiques that “the paper still offers little novelty”, but “could appeal to small number of researchers working on pigment cell differentiation”. We respectfully disagree with this outlook on the paper. We want to clarify again that our study is about a new paradigm in genomics, going beyond the general zebrafish community. In the past, time-consuming reverse genetics techniques were implemented to identify genes related to cell type differentiation. What we reveal through this work is that comparative epigenetics not only provide abundant gene candidates that impact cell differentiation, but also the key transcription factors that might drive cell fate decisions. Although we focus on the model of zebrafish pigment cell differentiation, we want to strongly emphasize that the strategy of performing comparative epigenetics to dissect cell fate decision is a practicable method that generates exciting hypothesis and testable candidates for validation. Indeed, this point was very well taken by Reviewer 1, who said “Jang et al. presents a substantial amount of meticulously collected and analyzed data about the epigenetic progression of pigment cell differentiation in zebrafish.” And “The data and the analysis are thorough and high quality. The conclusions are well-supported by the data. If anything, the authors have undersold their work.” In this manuscript, we highlight and validate alx4a to show strength of comparative epigenetics in dissecting zebrafish iridophore cell fate. We would like to emphasize again that this manuscript is not about comprehensively profiling the impact of a single transcription factor on iridophore development, but rather use alx4a as a proof-of-concept on the applicability of this under-utilized paradigm. In conclusion, we applied rigorous genomics screen (both in data and in analysis), made computational predictions, and validated the predictions by generating a novel, surprising, and interesting phenotype. We are regretful that Reviewer 3 seems to think the paper only appeals to researchers working on pigment cell differentiation in zebrafish. We respectively disagree. We hope that Reviewer 1’s comment highlights the impact of our work and how it appeals to a much broader readership, and that the Reviewer 1's considerations could receive considerable weight in the evaluation of this manuscript. Lastly, we want to address the additional experiment issues by referring to (2.2).*

Also, I find it odd that the data have not yet been submitted to GEO and that the code has not been made available in supplementary data or on a github page. The authors state: "Code used for analysis will be available upon request".

*(3.2) We apologize for not having the data available at the time of revision. Our standard practice is to upload the data to GEO once paper is accepted-in-principle. We have remedied this situation in the manuscript (lines 441-443): " All sequencing data presented in this paper has been deposited and publicly available through Gene Expression Omnibus (GEO) repository: GSE181287. The code used for the analysis in this manuscript is available at https://github.com/yjchen1201/zebrafish_pigment_cell_dev."*
